# Significance of *glioma-associated oncogene homolog 1 (GLI1)*expression in claudin-low breast cancer and crosstalk with the nuclear factor kappa-light-chain-enhancer of activated B cells (NFκB) pathway

**DOI:** 10.1186/s13058-014-0444-4

**Published:** 2014-09-25

**Authors:** Sierra A Colavito, Mike R Zou, Qin Yan, Don X Nguyen, David F Stern

**Affiliations:** 1 Department of Pathology and Yale Cancer Center, Yale University School of Medicine, New Haven, 06520 CT USA; 2 Department of Biology, University of Wisconsin La Crosse, La Crosse, 54601 WI USA

## Abstract

**Introduction:**

The recently identified claudin-low subtype of breast cancer is enriched for cells with stem-like and mesenchymal-like characteristics. This subtype is most often triple-negative (lacking the estrogen and progesterone receptors (ER, PR) as well as lacking epidermal growth factor 2 (*HER2*) amplification) and has a poor prognosis. There are few targeted treatment options available for patients with this highly aggressive type of cancer.

**Methods:**

Using a high throughput inhibitor screen, we identified high expression of glioma-associated oncogene homolog 1 (*GLI1)*, the effector molecule of the hedgehog (Hh) pathway, as a critical determinant of cell lines that have undergone an epithelial to mesenchymal transition (EMT).

**Results:**

High *GLI1* expression is a property of claudin-low cells and tumors and correlates with markers of EMT and breast cancer stem cells. Knockdown of *GLI1* expression in claudin-low cell lines resulted in reduced cell viability, motility, clonogenicity, self-renewal, and reduced tumor growth of orthotopic xenografts. We observed non-canonical activation of *GLI1* in claudin-low and EMT cell lines, and identified crosstalk with the NFκB pathway.

**Conclusions:**

This work highlights the importance of *GLI1* in the maintenance of characteristics of metastatic breast cancer stem cells. Remarkably, treatment with an inhibitor of the NFκB pathway reproducibly reduces *GLI1* expression and protein levels. We further provide direct evidence for the binding of the NFκB subunit p65 to the *GLI1* promoter in both EMT and claudin-low cell lines. Our results uncover crosstalk between NFκB and *GLI1* signals and suggest that targeting these pathways may be effective against the claudin-low breast cancer subtype.

**Electronic supplementary material:**

The online version of this article (doi:10.1186/s13058-014-0444-4) contains supplementary material, which is available to authorized users.

## Introduction

Breast cancer is a leading cause of cancer-related death in women. There are five major intrinsic breast cancer subtypes each with its own molecular characteristics, prognosis, incidence, and response to treatment [1]. Claudin-low tumors are mainly triple-negative invasive ductal carcinomas with a high frequency of metaplastic and medullary differentiation. There are conflicting reports as to the prevalence of these tumors, ranging from as low as 1.5% of tumors [2] to 5 to 14% of breast tumors [3]-[5]. Claudin-low tumor cells are enriched for characteristics of tumor-initiating cells and across a differentiation spectrum are most similar to mammary epithelial stem cells [5]. Claudin-low breast cancers are characterized by low expression levels of cell-cell adhesion molecules including E-cadherin and several of the tight junction claudin proteins, claudin 3, 4, and 7. This subtype is molecularly similar to cells that have undergone an epithelial-to-mesenchymal transition (EMT) and overlaps with the recently characterized mesenchymal and mesenchymal stem-like subclassifications of triple-negative breast cancer [6],[7]. Little is known about molecular therapeutic targets in this highly aggressive subtype of breast cancer.

EMT cells undergo a morphological transition from the epithelial polarized phenotype to the mesenchymal fibroblastoid phenotype. This process is marked by loss of cell-cell adhesion molecules, such as E-cadherin, downregulation of epithelial differentiation markers, and upregulation of mesenchymal markers. In cancer, it is hypothesized that EMT cells gain migratory potential at the expense of proliferative ability. EMT has therefore been implicated in the process of metastasis. There is a close association between the EMT core signature and the signatures that define the claudin-low and metaplastic breast cancer subtypes [7].

In vertebrates, canonical Hedgehog (Hh) pathway signal transduction occurs when one of the three ligands, Sonic, Indian, or Desert hedgehog, binds to the receptor Patched-1 (*PTCH1*) or its homolog, Patched-2. In the off-state, PTCH1 inhibits the activity of Smoothened (SMO). When stimulated by ligand this repression is lifted due to internalization and degradation of PTCH1. SMO then promotes the dissociation of the Suppressor of fused-Gli complex through an unknown mechanism. This allows for translocation of glioma-associated oncogene 1 (GLI1) and GLI family zinc finger 2 (GLI2) to the nucleus and degradation of the repressor form of GLI family zinc finger 3 (GLI3). In the nucleus, activated GLI proteins stimulate the transcription of Hh target genes, including *PTCH1* and *GLI1. PTCH1* is a Gli target, providing a negative feedback mechanism whereby the pathway is regulated. GLI1 is the key final output of the Hh pathway, and *GLI1* transcription is the most reliable marker of pathway activation [8]. The Hh pathway plays a critical role in vertebrate development, and is responsible for controlling cell fate, patterning, survival, proliferation and differentiation. In the adult organism Hh is active in the maintenance of stem cells [9]. Deregulation of this pathway can result in cancer.

There is evidence of a role for the Hh pathway in breast cancer. Some tumors exhibit loss of chromosomal regions containing *PTCH1* or amplification of regions containing *GLI1*[10], and Hh expression in the stroma is important [11]. Additionally, there is evidence for loss of *PTCH1* expression due to promoter methylation in human breast cancer, which correlated with decreased expression in samples from human ductal carcinomas *in situ* (DCIS) and in invasive ductal carcinomas [12]. Similarly, SMO has been found to be ectopically expressed in approximately 70% of DCIS samples, and 30% of invasive breast cancers [13]. Despite strong evidence for Hh pathway activation in breast cancer, overall few mutations in Hh pathway components have been identified [14]. *GLI1* is amplified in glioblastoma and has been implicated in other cancers. *GLI1* expression in mice causes mammary tumors with a basal-like phenotype [15]. Additionally, mammary stem cells are regulated by Gli transcription factors [16], and GLI1 has been associated with poorer outcome in ERα^-^ tumors [17] and overall [18],[19].

The nuclear factor kappa-light-chain-enhancer of activated B cells (NFκB) pathway plays a role in inflammation, cell survival, and transformation in response to stimuli including stress, cytokines, and microbial antigens. NFκB proteins are transcription factors, and inappropriate regulation of this family has been implicated in inflammatory and autoimmune diseases as well as cancer. Subunits of NFκB include v-rel avian reticuloendotheliosis viral oncogene homolog (Rel) family members RELA/p65, RELB, and c-REL, and NFκB subfamily members p105/p50 and p100/p52. NFκB family members associate with nuclear factor of kappa light polypeptide gene enhancer in B-cells inhibitor, alpha (IκBα), which sequesters them in the cytoplasm, and they are generally not active unless they dimerize with Rel subfamily members. NFκB has been implicated in the progression of breast cancer. For example, NFκB promotes cell migration and metastasis by upregulating expression of chemokine receptor CXCR4 [20]. CXCR4 is highly expressed in metastases from breast cancer patients, and is thought to play a role in homing of tumor cells to the bone marrow.

In order to identify possible drivers of proliferation in mesenchymal stem-like breast cancer, we conducted an inhibitor screen of human mammary epithelial cells (HMLE) induced to undergo an EMT. The results indicate the importance of GLI1 signaling in these cells, which further extended to a panel of claudin-low cancer cell lines. We identified non-canonical NFκB activation of *GLI1* in these cells, indicating crosstalk between GLI1 signaling and NFκB pathways in claudin-low and EMT breast cancer cells and suggesting a therapeutic route for claudin-low breast cancer.

## Methods

The experiments described did not include human subjects. All tumor data analyzed came from published expression datasets, which sought and obtained ethical approval and used Institutional Review Board approved protocols [5]. All animal studies were conducted in accordance with international, national, and university approved laws and policies. The animal studies received ethical approval from Yale University’s Institutional Animal Care and Use Committee.

### Cell culture

HMLE-EMT and control cell lines were a gift of Robert A. Weinberg (Massachusetts Institute of Technology, Cambridge, MA) and were propagated as previously described [21],[22]. MTSV1-7 lines were a gift from Joyce Taylor-Papadimitriou [23]. Claudin-low cell lines, BT549, HS578T, MDA.MB.157, MDA.MB.231, and MDA.MB.436 as well as MCF10a, were obtained from American Type Culture Collection (ATCC) and propagated according to instructions. All experiments were done on low-passage cells.

### High-throughput screen and dose-response curves

A total of 750 HMLE-shEcad cells per well were plated in 384-well plates in 20 μl of growth media and allowed to adhere overnight. The following day 10 nl of compounds from stock plates were added to each well (Table S1 in Additional file 1). The stock plates contained each agent at 16 concentrations from 10 mM to 0.3 nM. Seventy-two hours after drug addition, viability was assayed using CellTiter-Glo reagent (Promega, Madison, WI, USA). The protocol for the screen has been previously described by our laboratory [24].

For generating dose-response curves manually, 1,000 cells/well in 100 μl of media were plated in 96-well plates. Drug treatment and viability analysis was conducted as described above. Dose-response curves were generated using Graphpad Prism with Michaelis-Menten kinetics (Graphpad Software, Inc., San Diego, CA, USA).

### RNA isolation and real-time PCR

RNA was isolated using the RNeasy Plus kit (Qiagen, Germantown, MD, USA) and cDNA synthesized using the iScript kit (Bio-Rad Laboratories, Hercules, CA, USA) according to manufacturers’ protocols. Real-time PCR was performed on a Bio-Rad iCycler after combining the cDNAs with TaqMan universal PCR master mix and premixed FAM-labeled TaqMan probes (Applied Biosystems, Foster City, CA, USA). Abundance of mRNAs relative to *GAPDH* controls was calculated using the 2-ΔΔCt method.

### Cell lysis and immunoblotting

Lysates were prepared from subconfluent cells using NP40 lysis buffer (1.0% NP40, 150 mM NaCl, 50 mM Tris-HCl pH 7.4, 5 mM EDTA, 10% glycerol) with phophatase and protease inhibitors added. Lysates were transferred to polyvinylidine fluoride membranes and blocked in 5% nonfat milk in phosphate-buffered saline (PBS) with 0.1% Tween-20 (PBST). The primary antibodies used recognize GLI1, pEGFR, and ERBB2 (Cell Signaling Technology, Danvers, MA, USA) or epidermal growth factor receptor (EGFR), glyceraldehyde phosphate dehydrogenase (GAPDH) (Santa Cruz Technology, Santa Cruz, CA, USA). Membranes were washed with PBST and incubated with horseradish peroxidase-conjugated secondary antibodies. Gli1 blots were blocked in 5% bovine serum albumin (BSA).

### Gene expression analysis of tumors

Analysis of published expression data (Gene Expression Omnibus: GSE18229) from 337 mammary tumors and primary tissue was conducted using the UNC337 dataset [5]. The expression level of *GLI1* across the predefined subtypes was determined for each dataset following median centering using GEO2R. Results were analyzed by one-way analysis of variance (ANOVA).

### shRNA and retroviral infection

The following short hairpin (sh)RNA) sense sequences were cloned into the pSIREN-RetroQ (BD Biosciences, Franklin Lakes, NJ, USA) shRNA-expressing retroviral vector: *GLI1* #1: CCCAGATGAATCACCAAATTCAAGAGATTT [25] and #2: AAGCGTGAGCCTGAATCTGTG [26]. *RELA*: GCTGTGTTCACAGACCTGGCATCCGTCGA [27]. *NFKB1*: GCCAGAGTTTACATCTGA [28]. The negative control vector contains a scrambled sequence of a luciferase-directed shRNA (BD Biosciences).

For inducible shRNA-expressing viruses targeting *RELA*, the RHS4430-200223785 and RHS4430-200229897 GIPZ clones were purchased from Thermo Fisher Scientific (Waltham, MA, USA) and cloned in to pInducer10 via MluI and XhoI. Non-targeting sense sequence was 5′-GGATTCCAATTCAGCGGGAGCCTG-3′ [29]. Virus was produced by co-transfection of the pInducer10 constructs and packaging plasmids into 293 T cells. Virus was harvested and concentrated, and cells were infected and selected as described above. To induce expression of the hairpin, cells were treated with 1 μg/ml doxycycline for three days.

shRNA-expressing retroviruses were produced by co-transfection of the retroviral plasmids, pVSV-G and pCL-ECO (Clontech, Mountain View, CA, USA), into HEK 293 T cells (ATCC) using FuGene6 (Roche, Basel, Switzerland). Retrovirus was harvested in OptiMEM (Invitrogen) for five days, pooled, and concentrated with Centricon plus-20 columns (Millipore, Billerica, CA USA). Cells were infected at a multiplicity of infection of approximately five and selected in medium containing 0.6 μg/ml puromycin for at least three days before use.

### Proliferation assay

Cells infected with retrovirus were plated in 6-well dishes in medium containing 0.6 μg/ml puromycin. Every 24 hours, one well was trypsinized and counted using a Countess tissue culture counter (Invitrogen, Carlsbad, CA, USA).

### Migration assay

Retrovirally infected cells were selected for three days in medium containing 0.6 μg/ml puromycin, and plated in an 8.0 μm pore cell culture insert (BD Biosciences) in medium containing 1% fetal bovine serum (FBS), above medium containing 10% FBS. After 12 h, cells were scraped from the inside of the insert, and the insert was stained using Diff-Quik (Siemens, Erlangen, Germany). Cells in at least five fields of view were counted for each insert.

### Colony formation assay

Retrovirally infected cells were selected for three days in medium containing 0.6 μg/ml puromycin and plated at limiting dilutions in growth medium containing 0.6 μg/ml puromycin. Colonies were grown for 12 to 14 days, stained with Diff-Quik (Siemens), and counted under light microscopy.

### Sphere formation assay

A total of 40,000 cells were plated on 60 mM ultra-low attachment plates (Corning, Inc., Corning, NY, USA) in 4 mL of mammosphere growth medium (mammary epithelial basal medium (Lonza Group Ltd, Basel, Switzerland) with B27 supplement (Invitrogen), EGF (20 ng/mL), bFGF (20 ng/mL), heparin (7 μg/ml), and penicillin/streptomycin with 0.5% methylcellulose, adapted from [30]). Medium was replaced every 48 hours for 12 days, and spheres were counted under light microscopy.

For secondary sphere formation, primary sphere cultures were filtered using a 70 μm nylon cell strainer, to retain spheres of larger than 70 μm in diameter. Spheres were trypsinized until they dissociated to single cells. Cells were counted, and 20,000 cells were plated on the ultra-low attachment plates, following the same conditions as listed above for primary sphere formation.

### Flow cytometry

Cells were plated at 5 × 10^4^ cells/well in 6-well format and allowed to adhere overnight. The following day, cells were treated with 0.02 μM JK184 or 0.0002% DMSO vehicle control. After four days, floating cells were combined with adherent cells harvested by trypsinization and analyzed by flow cytometry using the BD Biosciences Pharmingen (San Diego, CA, USA) FITC Annexin V Apoptosis Detection Kit I. Samples were analyzed with the BD FACScaliburS flow cytometer with recording of 15,000 events per sample. Each line was treated independently and analyzed in three biological replicates. Gates were based on negative control signals, and plots generated using FlowJo 8.8.2 (Tree Star, Inc., San Carlos, CA, USA).

### Orthotopic xenograft studies

MDA.MB.436 cells with sh*GLI1* or control shRNA were trypsinized, washed twice in sterile 1 × PBS, and resuspended 1:1 in growth factor reduced matrigel (BD Biosciences). Cells were maintained on ice until injection. 2 × 10^6^ control (NT) cells were injected into the right fourth mammary fat pad of five-week-old female NOD/SCID mice (NOD.CB17-Prkdcscid/J, The Jackson Laboratory, Bar Habor, ME, USA). A contralateral injection was conducted, with 2 × 10^6^ sh*GLI1* cells injected into the left fourth mammary fat pad of each mouse.

Tumor growth was monitored using digital calipers, and tumor volume was calculated using the formula: W^2^ × L × 0.5, where L is the longer dimension and W the shorter. After seven weeks, animals were sacrificed and the tumors excised and weighed.

### Immunofluorescence

Cells were plated on glass chamber slides. The next day cells were washed twice with PBS and fixed in 2% paraformaldehyde with 0.1% Triton-X-100 in PBS for 15’ at room temperature (RT). Cells were washed twice in PBS, and quenched in 100 mM glycine in PBS for 5’ at RT. The slides were washed in PBS and permeabilized in 0.1% Triton-X-100 in PBS for 15’ at RT with humidification, followed by blocking in 5% BSA in PBST for 30’ at 37°C with humidification. Primary antibodies against p50 and p65 (Cell Signaling Technology) were diluted 1:250 with PBST and incubated overnight at 4°C with humidity. Slides were washed thrice with PBST, and incubated with Alexa Fluor 594-conjugated secondary antibody (Invitrogen) diluted 1:1000 in PBST for 1 h at RT. Slides were washed thrice with PBST and PBS, and mounted with Prolong Gold (Invitrogen).

### Chromatin immunoprecipitation (ChIP) assay

ChIP was performed according to published protocols with minor modifications [31]. Briefly, cells grown to 80% confluency were fixed in 1% formaldehyde for 10’ at RT, followed by 0.125 M glycine quench. Plates were rinsed twice with PBS, and adherent cells were scraped in ice-cold PBS and collected by centrifugation. Cells were lysed on ice in buffer with protease inhibitors (5 mM PIPES pH 8, 85 mM KCl, 1% v/v igepal) using a dounce homogenizer. After centrifugation, the nuclei pellet was lysed on ice in buffer containing protease inhibitors (50 mM Tris-HCl pH 8, 10 mM EDTA, 1% w/v SDS). The chromatin was sonicated on ice for 15’ with a 30″-on/30″-off cycle using a Biorupter UCD-200 (Diagenode Inc., Denville, NJ, USA) set to high. The chromatin was cleared by centrifugation, and for each ChIP from 10^7^ cells was diluted to 1 mL with buffer containing protease inhibitors (50 mM Tris-HCl pH 7.4, 150 mM NaCl, 1% v/v igepal, 0.25% w/v deoxycholic acid, 1 mM EDTA, pH 8). Antibody was added (Histone H3, p65, and IgG from Cell Signaling Technology) and incubated at 4°C overnight on a rotating platform.

A total of 30 μl of magnetic protein G beads (Cell Signaling Technology) were added to each ChIP, and incubated on a rotating platform for 2 h at 4°C. Beads were washed twice with dilution buffer, three times with 100 mM Tris-HCl pH 9, 500 mM LiCl, 1% v/v igepal, 1% w/v doxycholic acid, and once with 100 mM Tris-HCl pH 9, 500 mM LiCl, 150 mM NaCl, 1% v/v igepal, 1% w/v doxycholic acid. Antibody/chromatin complexes were eluted in 100 μl buffer (50 mM NaHCO_3_, 1% w/v SDS) for 1 h at RT. The samples were adjusted to 0.54 M NaCl, and incubated at 67°C overnight to reverse the crosslinks.

Samples were treated with RNaseA, and purified using a PCR cleanup kit (Qiagen). Real-time PCR was conducted using iQ SYBR Green Supermix (Bio-Rad Laboratories) on a CFX96 Real-Time System thermal cycler (Bio-Rad Laboratories) with 15 μl reactions, in triplicate. The following primer sets were used: *GLI1* site 1: 5′-GGGTAAGGGCTGTTGAGGTA-3′, 5′-AAATGCTTGTCTCCCAGTGG-3′; 2: 5′-GAGCTAGGATGTGGGAGGTC-3′, 5′-TGAGAAACGGAGAGGCAGAG-3′; 3: 5′-AGGGTCGGAATAAGTGTGGT-3′, 5′-GTGTGTATGGGGAGGAGGAG-3′; 4: 5′-CTCCTCCTCCCCATACACAC-3′, 5′-CTCTCAGCACATCCGGAAAG-3′; 5: 5′-ACGCCATGTTCAACTCGATG-3′, 5′-GAGATCTGCCAAATCCTCAAGG-3′; 6: 5′-GCCCAATCCTTCCTGAGACT-3′, 5′-CGGGCAGAGTCATGGGGA-3′; GAPDH: 5′-TACTAGCGGTTTTACGGGCG-3′, 5′-TCGAACAGGAGGAGCAGAGAGCGA-3′.

## Results

### EMT cells are more sensitive to GLIinhibitor

**Figure 1:**
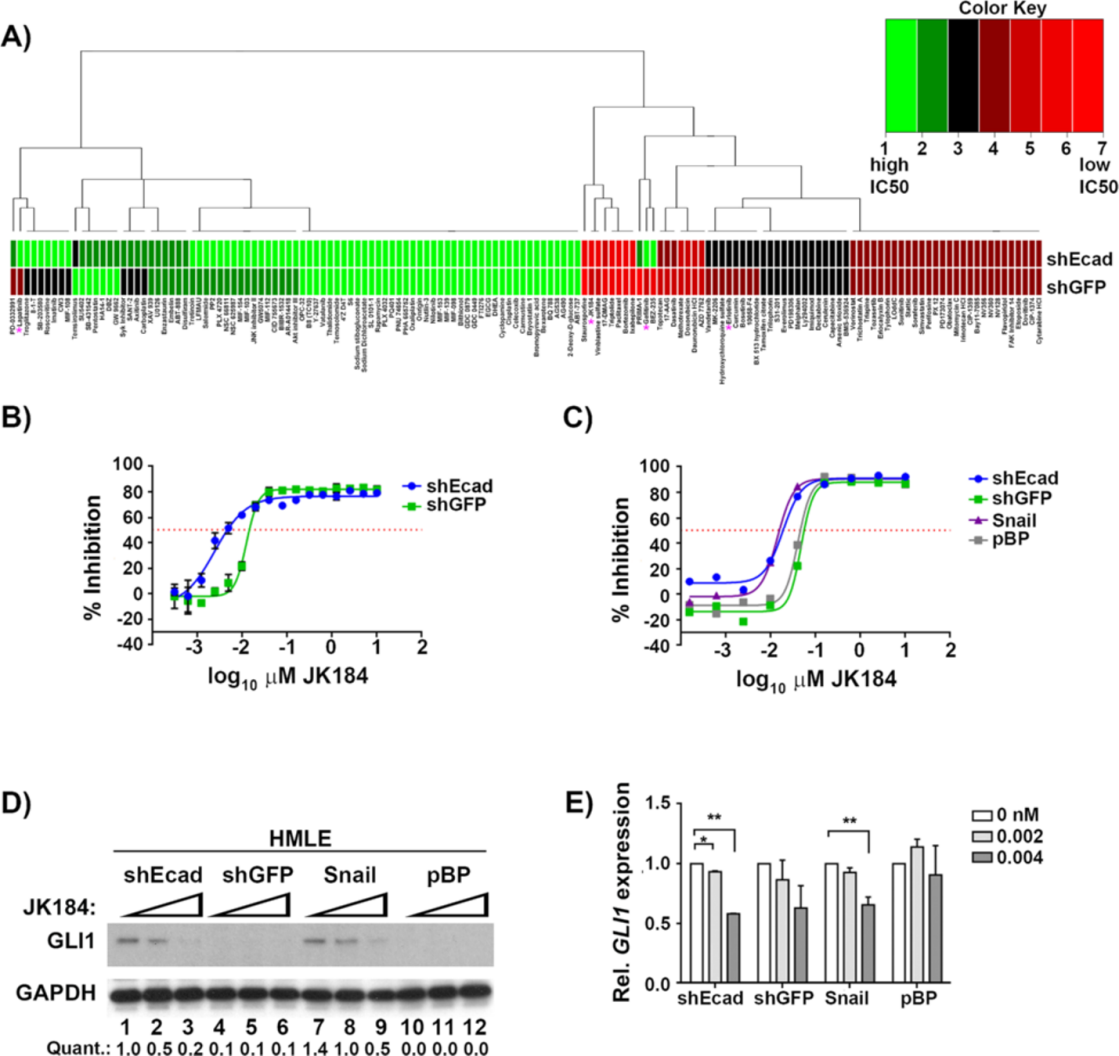
**JK184 is more effective at inhibiting EMT cell proliferation. (A)** Clustering of Spearman ranked data of inhibition concentration (IC) values for the inhibitor screen. Rank values are as follows for μM concentrations: 1: no IC values are between 0 and 10; 2: IC_10_ between 0 and 10, and IC_25_ not between 0 and 10, and IC_50_ not between 0 and 10; 3: IC_25_ between 0 and 10, and IC_50_ not between 0 and 10; 4: IC_50_ between 0.1 and 10; 5: IC_50_ between 0.01 and 0.1; 6: IC_50_ between 0.001 and 0.01; 7: IC_50_ < 0.001. Asterisks mark the agents mentioned in the text. **(B)** Dose-response curve for JK184 treatment of HMLE-shEcad cells (blue) and HMLE-shGFP cells (green). Error bars indicate coefficient of variance between triplicate treatment. **(C)** Dose-response curve for JK184 treatment of HMLE-shEcad cells (blue), HMLE-shGFP cells (green), HMLE-Snail cells (purple), and HMLE-pBP cells (gray). Treatment was conducted in 96-well plates. **(D)** Immunoblot showing response of *GLI1* expression in HMLE cells with JK184 drug treatment. Doses were 0, 0.002, and 0.004 μM JK184 for 72 h. GAPDH serves as a loading control. *GLI1* quantification levels were normalized to HMLE-shEcad untreated levels, and appear below the lane numbers in the figure. **(E)** Real-time PCR data showing relative *GLI1* mRNA transcript levels from cells treated as in **D)**. Error bars represent standard error between two independent experiments. * = *P* ≤0.05, *t* test. ** = *P* ≤0.005, *t* test. GAPDH, glyceraldehyde phosphate dehydrogenase; GLI1, glioma-associated oncogene 1; HMLE, human mammary epithelial cells, transformed with large T and small t antigens.

HMLE-shEcad cells are HMLE with E-cadherin knockdown [21], which results in EMT associated with mesenchymal-like and stem-like characteristics [7],[22]. In order to identify possible drivers of proliferation, these cells were assayed for growth inhibition by a panel of 150 compounds at 16 doses each. Overall, the HMLE-shEcad cells were more resistant to this panel of inhibitors than were the control HMLE-shGFP cells (Figure 1A and Table S1 in Additional file 1). In every case where the response between the two cell lines differed by an IC_50_ value of more than 0.01 μM for standard of care therapies for triple-negative breast cancer, the HMLE-shEcad cell lines had a poorer response (higher IC_50_ value) than the control cell line.

Interestingly, epidermal growth factor receptor (EGFR) inhibitors erlotinib and gefitinib profoundly inhibited the proliferation of the control cell lines, while the EMT cell lines were resistant to this treatment, with neither EGFR inhibitor approaching 50% growth inhibition at concentrations up to 10 μM (Figure S1A-C in Additional file 1). In follow-up studies we observed that despite having an intact EGFR pathway, HMLE-shEcad cells lack high levels of baseline phosphorylated EGFR, and have lower levels of EGFR overall, possibly rendering them immune to EGFR-inhibitor treatment, and suggesting that the cells do not rely on EGFR signaling for survival. ERBB2 levels do not differ between control or EMT cells (Figure S1D-F in Additional file 1). The lack of response to EGFR inhibition is in concordance with the recent finding that activation of the EMT program effects a shift from EGFR to platelet-derived growth factor receptor (PDGFR) signaling [32].

**Figure 2:**
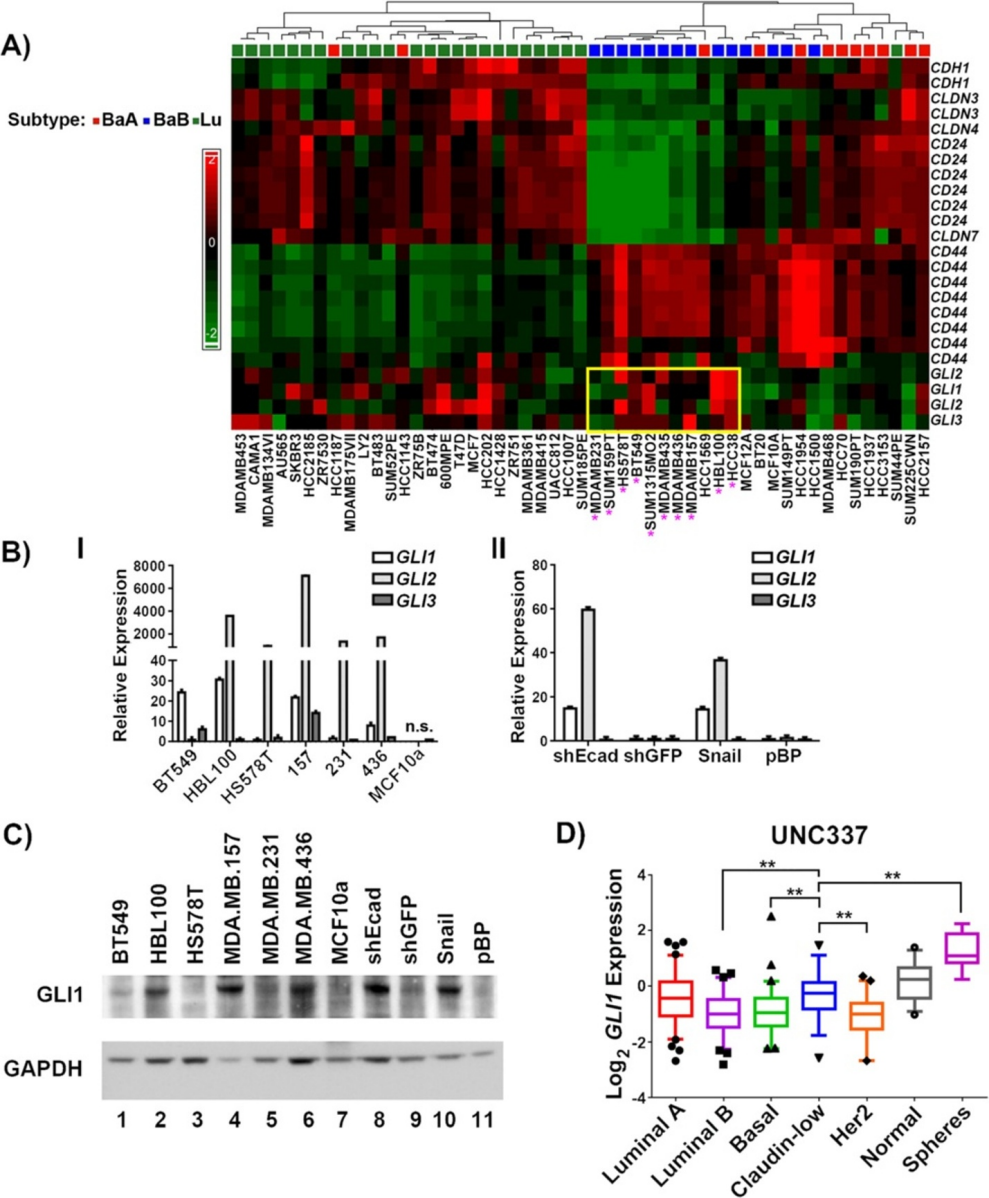
**Claudin-low cell lines express higher transcript and protein levels of** ***GLI1*** **. (A)** Expression of *CD44*, *CD24*, *CDH1*, *GLI*, and *CLDN3*, *4*, and *7*, transcripts across a panel of breast cancer cell lines [34]. Multiple probes for each gene were extracted from microarray data and their expression values were Z transformed and plotted as a heat map. BaA = basal A, BaB = basal B, Lu = luminal. Asterisks denote cell lines originally identified as claudin-low [5]. Yellow box surrounds the expression of Gli family members in claudin-low cell lines. **(B)** Graph of relative Gli family expression levels in claudin-low (I) and EMT (II) cell lines, relative to controls. Error bars represent standard error, and n.s. indicates that no signal was achieved under the parameters used. **(C)** Immunoblot of GLI1 levels in the indicated claudin-low cell lines. GAPDH serves as a loading control. **(D)** Plot of *GLI1* expression data from UNC337 mammary tumor and tissue dataset, based on subtype. 99% confidence interval is shown, with outliers plotted as single data points. Significance was calculated using one-way ANOVA using multiple comparisons, and all significant comparisons between the claudin low data set and others are indicated. ** = *P* ≤0.005. ANOVA, analysis of variance; EMT, epithelial-to-mesenchymal transition; GAPDH, glyceraldehyde phosphate dehydrogenase; GLI1, glioma-associated oncogene 1.

While the HMLE-shEcad cells were more resistant overall to the panel of inhibitors, there were a few compounds that selectively inhibited the growth of the EMT cells compared to controls. One such agent was the GLI1 inhibitor JK184 [33], which was more active against HMLE-shEcad cells than controls. The IC_50_ value for JK184 was 3.5-fold lower for the HMLE-shEcad cells compared to the control HMLE-shGFP cell line (0.004 μM compared to 0.014 μM, Figure 1B). The dose-response curve for the HMLE-shEcad cells was shifted to lower doses compared to the HMLE-shGFP curve, indicating a concentration window between 0.001 to 0.1 μM in which JK184 is more effective on the EMT cells. This selective sensitivity to the GLI1 inhibitor was confirmed in another EMT cell line, HMLE-Snail [22], which was similarly more sensitive to JK184 compared to HMLE-pBP control cells (Figure 1C). Immunoblot and real-time PCR analysis indicated that both EMT cell lines have higher protein and transcript levels of *GLI1* than controls, as well as elevated levels of *GLI2*, while the extent of *GLI3* expression was consistent across the cell lines (Figures 1D and 2B).

Treatment of both EMT cell lines with the IC_25_ and IC_50_ doses of JK184 yielded a dose-dependent decrease in *GLI1* protein and transcript levels (Figure 1D-E). GLI1 positively regulates its own transcription [34], so it is likely that the JK184-dependent decrease in *GLI1* transcript and protein levels arises from decreased activity of the JK184 target GLI1. *GLI2* and *GLI3* are not direct targets of GLI1, and neither *GLI2* nor *GLI3* transcript levels were affected in JK184-treated HMLE cells (Figure S2A-B in Additional file 1).

### Claudin-low cell lines express elevated levels of Gli proteins and are more sensitive to the GLIinhibitor

We next determined if Gli expression is associated with a particular breast cancer subtype. Using a previously published dataset, cell lines were clustered based on CD24, CD44, E-cadherin (CDH1), and CLDN3, 4, and 7 expression status [35]. This reveals a subgroup of basal B cell lines identified as claudin-low [5],[36]. Claudin-low tumors express decreased levels of *CDH1*, are enriched for EMT markers, and are CD24 low/CD44 high, which are features of self-renewing breast cancer stem cells [5].

Within the basal subtype, the claudin-low cells generally express higher levels of *GLI1*, *GLI2*, and *GLI3* (Figure 2A). We confirmed this expression data in several claudin-low cell lines, and found that similarly to the EMT cell lines, claudin-low cells express higher transcript levels of the Gli family of transcription factors than do MCF10a cells, an immortalized but non-tumorigenic mammary cell line (Figure 2B). Additionally, claudin-low cell lines have higher protein levels of GLI1 than do MCF10a cells (Figure 2C).

Given the elevated expression levels of *GLI1* we observed in claudin-low cell lines, we determined if claudin-low tumors display similarly elevated *GLI1* levels. An analysis of a published dataset of over 330 tumors, including 37 claudin-low tumors, revealed elevated *GLI1* expression levels in claudin-low tumors compared to basal tumors (*P* = 0.001, Figure 2D). Some luminal A tumors express *GLI1*, which mirrors the expression data for the cell lines (Figure 2A).

**Figure 3:**
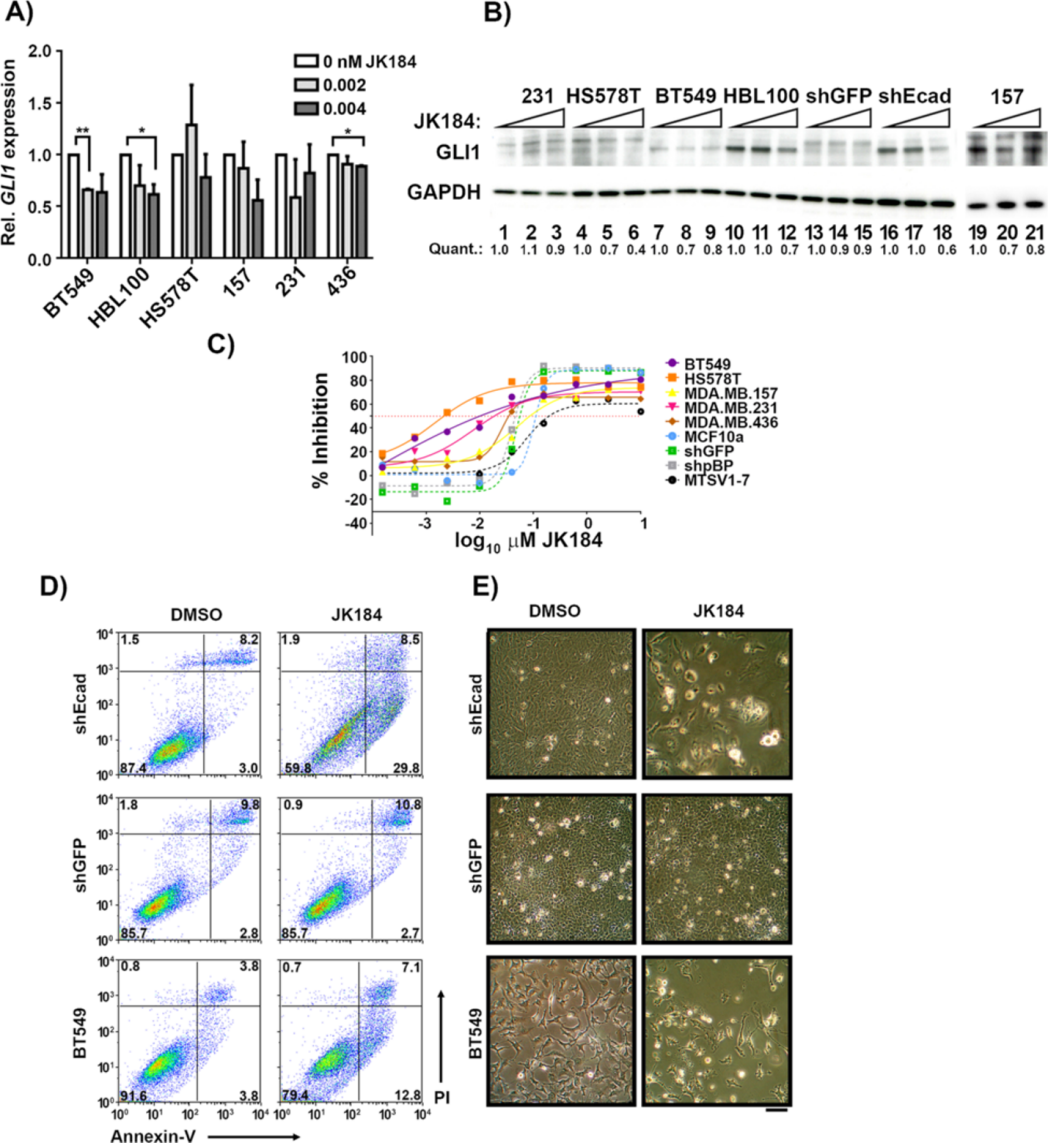
**JK184 inhibits growth of claudin-low cell lines. (A)** Real-time RT-PCR analysis of Gli1 RNA expression in JK184-treated claudin-low cell lines. Doses were 0, 0.002, and 0.004 μM JK184 for 72 h. Values were normalized to DMSO vehicle control, error bars are the standard error between two independent experiments. * = *P* ≤0.05, *t* test. ** = *P* ≤0.005, *t* test. **(B)** Western blot showing protein expression levels in claudin-low cells treated as in **A)**. Lanes 19 to 21 are from a separate gel. **(C)** Dose-response curve for 72 h JK184 treatment of claudin-low and MCF10a, HMLE-shGFP, HMLE-pBP, and MTSV1-7 cell lines. **(D)** Flow cytometric analyses of cells treated with JK184 (0.02 μM) for four days followed by staining with Annexin-V and propidium iodide (PI). Images representative of three independent experiments. **(E)** Light microscopy images of cells treated as in **A)**. Scale bar indicates 100 μm.

Since claudin-low cell lines and tumors preferentially express *GLI1* transcripts, and claudin-low cells are transcriptionally similar to the JK184-sensitive EMT cells, we determined the dose-sensitivity of claudin-low cell lines to JK184. Overall, claudin-low cell lines are more sensitive to JK184 treatment than are MCF10a, MTSV1-7, or HMLE-shGFP and HMLE-pBP cells, and JK184 induced a dose-dependent decrease in *GLI1* transcript and protein levels in these cells (Figure 3A-C). *GLI2* and *GLI3* levels were not significantly altered with JK184 treatment (Figure S2C-D in Additional file 1). Taken together, these data establish similar patterns of GLI1 signaling and GLI1 inhibitor sensitivity in EMT and claudin-low cell lines, providing further evidence for the similarity of EMT and claudin-low cells.

### GLIinhibitor treatment results in increased apoptosis

Since JK184 treatment reduced cell accumulation, we identified whether there is an elevated rate of apoptosis in JK184-treated cells. Treatment with the IC_50_ dose of JK184 enhanced the proportion of HMLE-shEcad cells that stained with Annexin-V, but were negative for propidium iodide (PI) (*P <*0.0001, *t* test; Figure 3D). This staining pattern is consistent with early-apoptotic cells, while PI-positive or dual-stained cells indicate apoptosis and/or necrosis. JK184-treated cells were sparser and less well spread than vehicle-treated counterparts (Figure 3D). JK184 treatment did not significantly affect the staining of HMLE-shGFP cells or their morphology (Figure 3D). Over four days of treatment there was increased cell death of the BT549 cells, as increased Annexin-V and PI staining were observed in treated cells, and these cells appeared rounded up compared to vehicle-treated counterparts (*P* = 0.001, *t* test; Figure 3D).

### Knockdown of GLIdecreases proliferation of claudin-low cell lines

**Figure 4:**
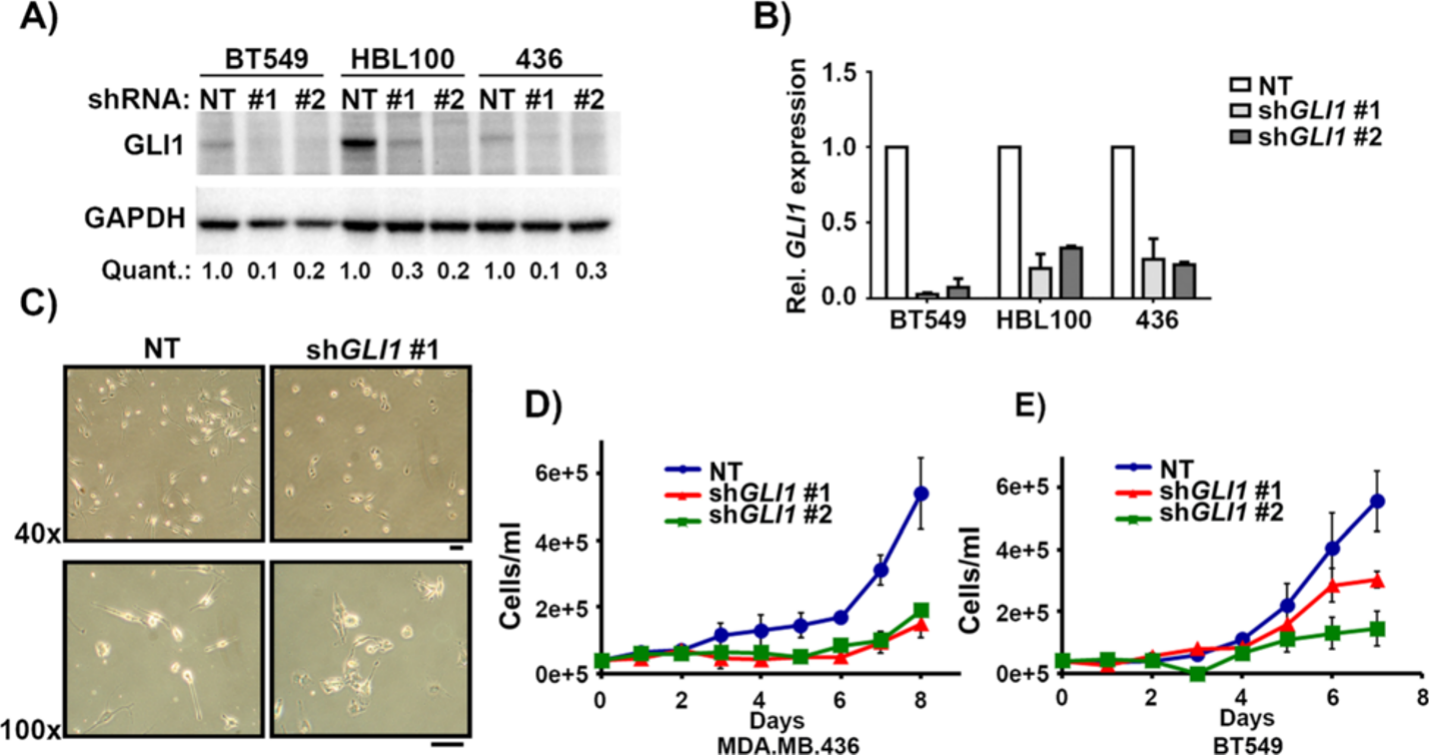
**Knockdown of** ***GLI1*** **decreases proliferation of claudin-low cell lines. (A)** Western blot of cells infected with retrovirus expressing either non-targeting shRNA (NT), or shRNA targeted against *GLI1* (#1, #2). Cells were infected, and selected for three days prior to blotting. GAPDH serves as a loading control. Quantification is relative to NT for each cell line. Blot is representative of three independent experiments. **(B)** Plot of *GLI1* transcript levels in response to the shRNAs and treatment described in **A)**. Error bars represent the standard error between three independent experiments. **(C)** Light microscopy images of MDA.MB.157 cells infected with either non-targeting (NT) or *GLI1* -directed shRNA. Scale bars indicate 100 μm. **(D, E)** Plot detailing the proliferation of MDA.MB.436 cells **(D)** or BT549 cells **(E)** infected with the indicated retroviruses and grown in selection media. GAPDH, glyceraldehyde phosphate dehydrogenase; GLI1, glioma-associated oncogene 1; sh, short hairpin.

Given the markedly elevated *GLI1* levels observed in the claudin-low subtype, we wanted to determine the biological importance of GLI1 in claudin-low cell lines. We investigated the effects of *GLI1* knockdown on aggressive characteristics of claudin-low tumor cells, including proliferation, migration, and anoikis. GLI1 has been implicated in several of these processes in transformed cells [37] and in some breast cancer cell lines [38]. Since there did not always appear to be a direct correlation between expression levels of *GLI1* and sensitivity to JK184 treatment, and to avoid possible off-target effects of JK184, we utilized a specific genetic approach to reduce *GLI1* expression. Two different shRNA viruses both induced stable, specific knockdown of *GLI1* protein (Figure 4A) and transcript levels (Figure 4B). Neither *GLI2* nor *GLI3* transcript levels were affected by *GLI1* knockdown (Figure S2E-F in Additional file 1). Knockdown of *GLI1* in MDA.MB.157 cells resulted in cells that appeared rounder and less elongated compared to cells with control knockdown, which maintained the long spindle shape characteristic of mesenchymal cells (Figure 4C). MDA.MB.157 cells have an extended doubling time of greater than 60 hours, so we therefore evaluated the biological effects of *GLI1* knockdown in two other claudin-low cell lines with high *GLI1* expression, BT549 and MDA.MB.436 cells. Knockdown of *GLI1* greatly reduced the growth rates of these cell lines compared to cells expressing the non-targeting shRNA (Figure 4D-E). Proliferation of MCF10a cells, which do not express endogenous *GLI1*, was not significantly affected by infection with the sh*GLI1* retrovirus (Figure S3F in Additional file 1). The proliferation of the immortalized human mammary epithelial cell line MTSV1-7 was significantly, though slightly, impaired following *GLI1* knockdown. These cells do express some *GLI1* (Figure S3A-C, F in Additional file 1).

### Loss of GLIreduces cell migration and anchorage-independent growth

**Figure 5:**
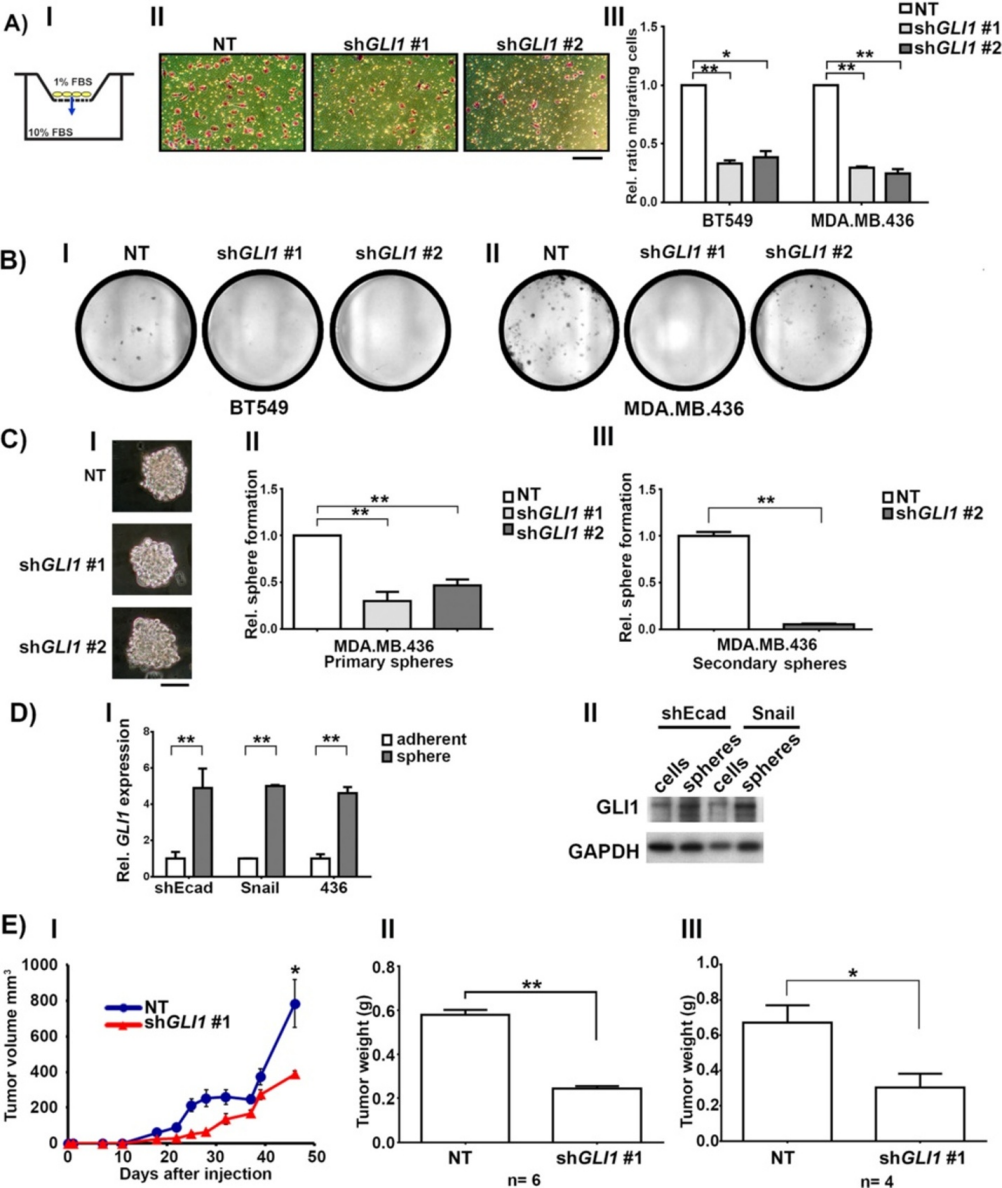
**Decrease in** ***GLI1*** **expression inhibits cell migration and anchorage-independent growth. (A)** Panel I: Cartoon depicting experimental setup. Cells (yellow) were plated in the top of a Boyden chamber in 1% FBS above a lower-chamber of media containing 10% FBS. After 16 hours the number of cells that have migrated to the bottom side of the filter were stained and counted. II: Light microscopy images of the bottom side of the migration filter after staining. BT549 cells were infected with the indicated retroviruses and selected prior to experimentation. Scale bar indicates 100 μm. III: Migration of BT549 and MDA.MB.436 cells infected with the indicated retroviruses and assayed as described in I. **(B)** Clonogenic colony formation assay in BT549 (I) and MDA.MB.436 (II) cells. **(C)** Panel I: Representative 12 day spheres formed in MDA.MB.436 cells infected with the indicated retroviruses. Scale bar indicates 100 μm. II: Relative sphere formation by MDA.MB.436 cells. **(D)** Panel I: *GLI1* mRNA expression obtained from real-time RT-PCR analysis of mRNA from adherent cells incubated in mammosphere medium (white bars) or sphere cells grown under non-adherent conditions (gray bars). II: Western blot detailing GLI1 levels in HMLE-shEcad or HMLE-Snail adherent and sphere cells. For all: * = *P* ≤0.05, *t* test. ** = *P* ≤0.005, *t* test. **(E)** (I) Plot of tumor volume over time arising from orthotopic injection of MDA.MB.436 cells infected with either non-targeting (NT) or *GLI1* knockdown constructs. n = six animals. (II) Final averaged tumor weights at seven weeks post-injection for n = six animals. (III) Final averaged tumor weights from an independent biological replicate of the orthotopic xenograft study, conducted on four animals. FBS, fetal bovine serum; GLI1, glioma-associated oncogene 1.

*GLI1* knockdown significantly reduced the transwell migration of BT549 and MDA.MB.436 cells in response to an FBS gradient with both *GLI1* knockdown constructs used (Figure 5A). The low FBS in the top chamber of the transwell (1%) and the relatively short assay time (12 h) minimized the impact of reduced proliferation resulting from *GLI1* knockdown. MCF10a cells were not affected (Figure S3E in Additional file 1).

In clonogenicity assays, knockdown of *GLI1* reduced colony formation of BT549 and MDA.MB.436 cells (Figure 5B), whereas MCF10a cells were largely unaffected (Figure S3D in Additional file 1). Mammospheres are derived from mammary cells grown under non-adherent conditions. They are enriched in early progenitor/stem cells and are able to differentiate along all three mammary epithelial lineages [30]. The sphere-initiating subset of mammary cells have cancer stem cell-like characteristics, including the ability to self-renew and to differentiate into mature mammary cells that lack stem cell features [16],[30]. We did not observe any significant sphere formation resulting from either the control HMLE-shGFP or HMLE-pBP cell lines, as has been previously reported [22]. We also did not achieve significant levels of spheres resulting from BT549 cells, despite their tumorigenicity [39]. Therefore, we first investigated the effects of *GLI1* knockdown on the sphere-forming ability of MDA.MB.436 cells. *GLI1* knockdown reduced primary and secondary sphere formation by MDA.MB.436 cells (Figure 5C). Secondary sphere formation is a measure of the self-renewal capacity of the sphere-forming cells. The ability of the cells to form secondary spheres was enhanced compared to primary sphere forming ability, producing on average 454 secondary spheres from 20,000 plated cells (0.055%), compared to the average primary sphere forming ability of 254 spheres from 40,000 plated cells (0.006%). These data, combined with the higher *GLI1* transcript and protein levels we observed in spheres derived from EMT and/or claudin-low cells compared to control adherent cells (Figure 5D) indicates that *GLI1* is important for formation of spheres. Elevated *GLI1* levels were seen in primary mammospheres formed from normal and tumorigenic breast tissue from a published dataset (Figure 2D). Higher levels of *GLI1* transcripts in mammospheres compared to differentiated mammary cells has been reported [16],[40], but we observed that *GLI1* levels in mammospheres are even higher than those in EMT cells grown under adherent conditions.

We also wanted to investigate the effects of *GLI1* knockdown in a basal cell line, HCC1806, which expresses elevated levels of *GLI1* (Figure S3A-C in Additional file 1). Significantly fewer spheres resulted from cells in which *GLI1* expression was reduced compared to controls (Figure S3G in Additional file 1). Additionally, similarly as to seen with the MDA.MB.436 cells and the EMT cell lines, there was significantly more *GLI1* expression from HCC1806 cells grown as spheres compared to those grown under adherent conditions (Figure S3H in Additional file 1). This is further evidence that *GLI1* is activated and functionally important in sphere-initiating and/or early progenitor cells across a spectrum of cell lines.

### GLIexpression is important for tumor formation in vivo

Having shown that *GLI1* is essential for the aggressive and stem-like characteristics of claudin-low cell lines *in vitro*, so we next examined the effects of *GLI1* knockdown *in vivo*. While previous studies have highlighted the ability of ectopic *GLI1* expression to form tumors *in vivo*[15],[40], we wanted to investigate the effects of *GLI1* knockdown on the tumor-forming abilities of claudin-low MDA.MB.436 cells as orthotopic xenografts. While all sh*GLI1* expressing cells eventually formed tumors, *GLI1* knockdown tumors were smaller on average than tumors resulting from contralateral control knockdowns (Figure 5E, panel I and II). Similar results were seen for an independent biological replicate (Figure 5E, panel III, and Figure S4 in Additional file 1). These data are consistent with reports from other groups, in which knockdown of *GLI1* delayed onset of tumor formation from injection of claudin-low SUM1315 cells [40], and reduced the incidence of metastasis to the lung by claudin-low MDA.MB.231 cells [41].

### The NFκB pathway contributes to elevated GLIlevels in claudin-low and EMT cells

**Figure 6:**
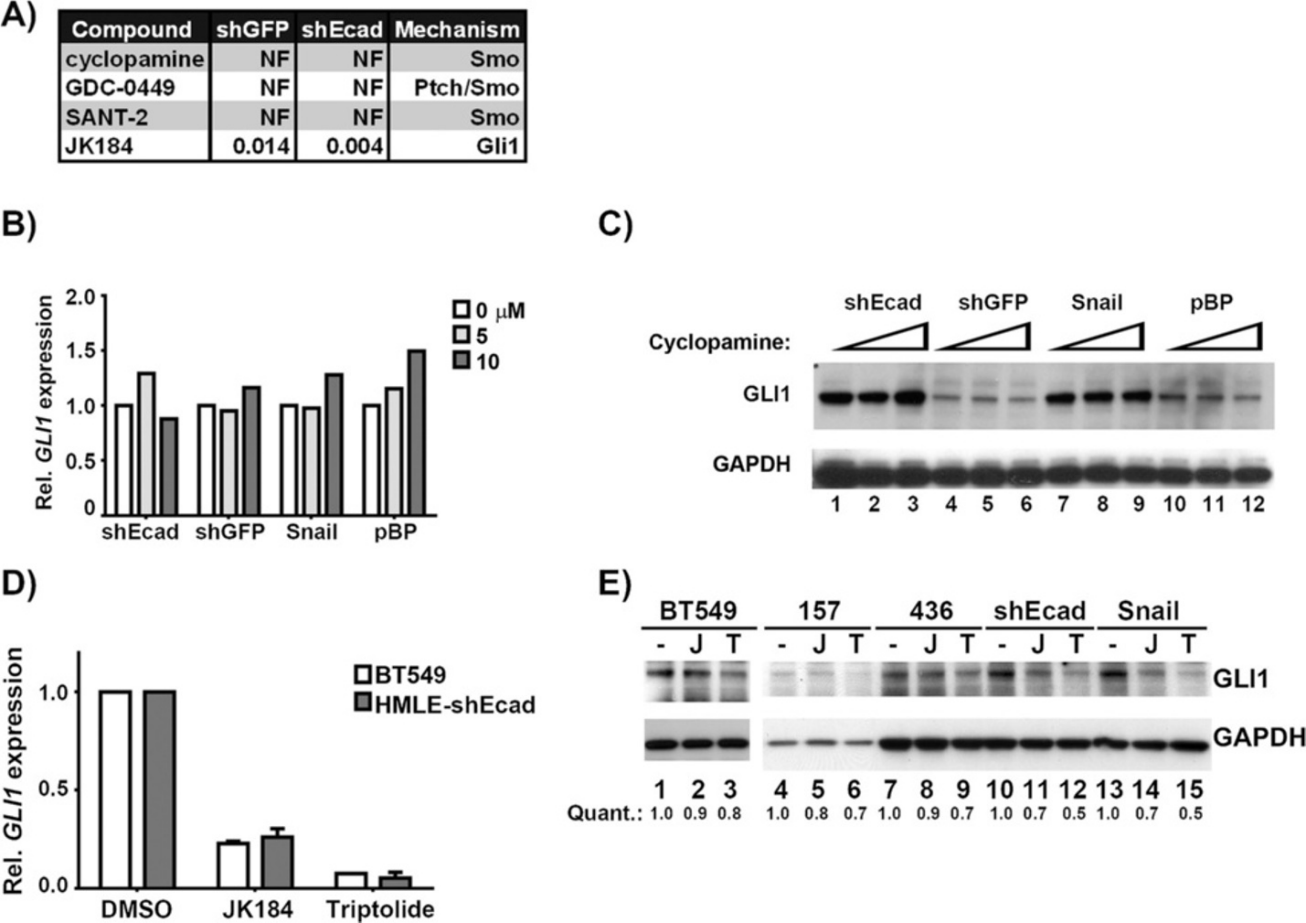
**EMT and claudin-low cells are insensitive to Hedgehog (Hh) pathway inhibitors. (A)** Inhibition concentration (IC)_50_ values obtained with other Hh pathway antagonists from the 384-well screen. NF indicates not determined, either because no curve fit could be obtained, or the IC_50_ was outside the experimental concentrations tested. **(B)** *GLI1* transcript levels in HMLE-shEcad and HMLE-shGFP cells treated with cyclopamine (0, 5, or 10 μM for 72 hours). **(C)** Immunoblot of GLI1 in HMLE cells treated with cyclopamine as in **B)**. **(D)** Gli1 transcript levels after 16 h treatment with 1 μM JK184 or triptolide. Error bars indicate the standard error of two independent experiments. * = *P* <0.05, *t* test. ** = *P* <0.005, *t* test. **(E)** Anti-GLI1 immunoblot following treatment described in **A)**. Lanes 1 to 3 are from a separate gel. Quantification is normalized to no treatment condition. EMT, epithelial-to-mesenchymal transition; GLI1, glioma-associated oncogene 1; HMLE, human mammary epithelial cells, transformed with large T and small t antigens.

Whereas JK184 reduced *GLI1* levels, cyclopamine, which inhibits the Hh pathway through SMO, did not affect proliferation or *GLI1* levels in EMT cells (Figure 6A-C). Additionally, two other SMO inhibitors in our screen had no effect on accumulation of EMT cells (Figure 6A). The ineffectiveness of cyclopamine on *GLI1* expression is consistent with published results for breast cancer cell lines including SUM149, T47D, MCF7, and MDA.MB.231 [38],[40],[42]. Taken together, these data indicate that the elevated *GLI1* levels in EMT and claudin-low cell lines are not caused by classical activation of the pathway through SHH-PTCH1-SMO. Non-canonical activation of the Hh pathway can occur with activation of the Gli family, mainly *GLI1*, by MAPK/ERK [43], AKT [44], KRAS [45], mTOR/S6K1 [46], and more recently NRP2 [40]. The mechanisms of SMO-independent upregulation of *GLI1* are poorly understood.

To determine which upstream pathways are responsible for elevated *GLI1* in claudin-low and EMT cells, we screened several candidate molecules for reduction of *GLI1* expression (Figure S5A-B in Additional file 1). Triptolide, an NFκB inhibitor [47]-[49] reduced *GLI1* transcript and protein levels in both HMLE-shEcad and BT549 cell lines (Figure 6D-E). The activity of triptolide as an NFκB inhibitor was confirmed by means of a reporter assay (Figure S5C in Additional file 1).

**Figure 7:**
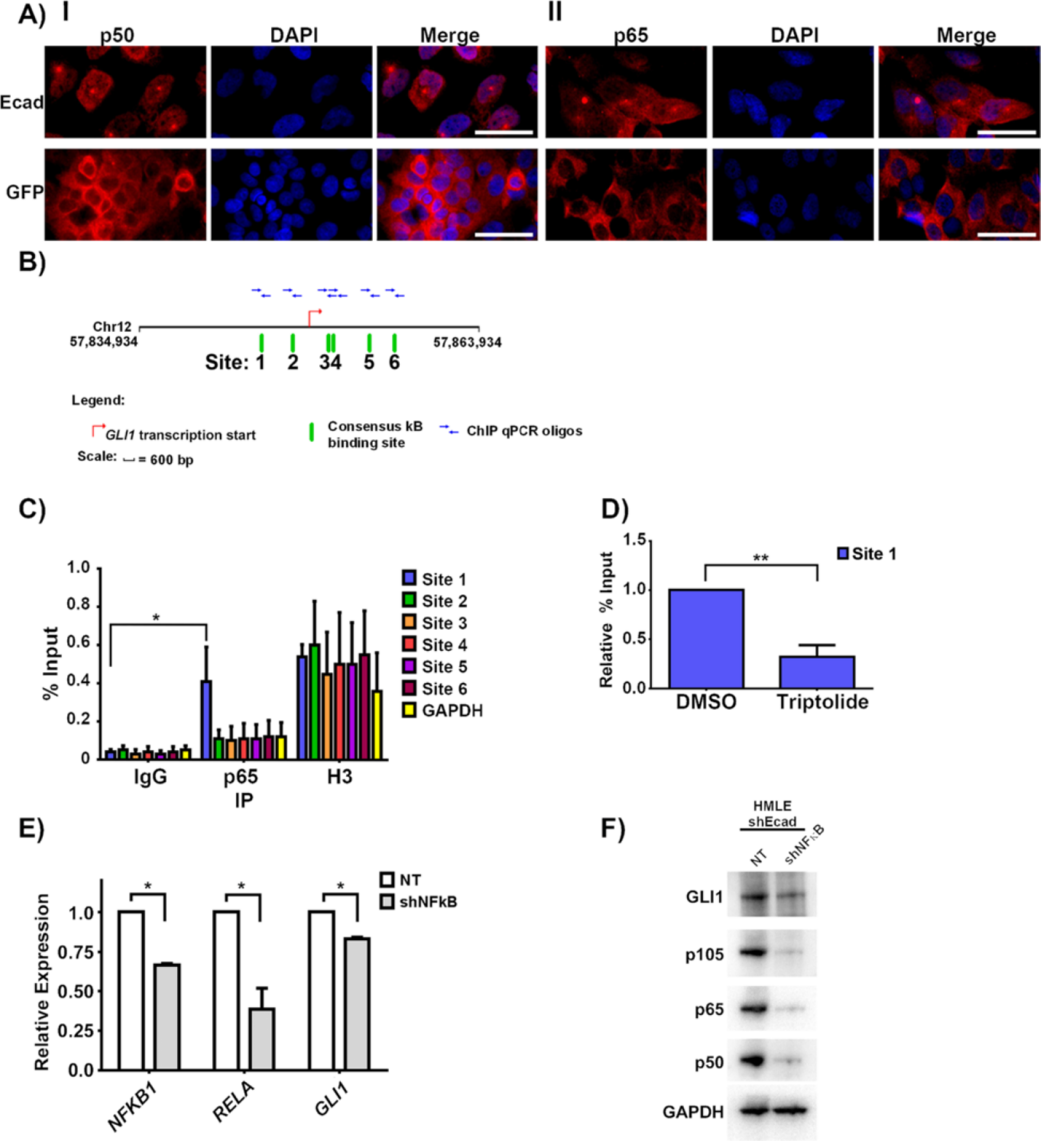
**Crosstalk between NFκB and GLI1 signaling pathways. (A)** Immunofluorescence images showing localization of NFκB p50 (Panel I) or p65 (Panel II) subunits in HMLE-shEcad (top row) or HMLE-shGFP (lower row) cells. Scale bar indicates 50 μm. **(B)** Map of putative NFκB binding sites surrounding the *GLI1* promoter. Red arrow marks transcription start site. Green lines indicate sequences matching the consensus κB binding site; putative sites are numbered 1 to 6. Blue arrows indicate primer sets used for ChIP. **(C)** ChIP of HMLE-shEcad cells with indicated antibodies. Error bars indicate standard error among three independent experiments. **(D)** NFκB p65 ChIP to the *GLI1* promoter (site 1) following six-hour treatment with 1 μM triptolide. Graph was normalized to binding with vehicle control (DMSO). Error bar indicates standard error among three independent experiments. **(E)** Real-time PCR data showing *RELA*, *NFKB1*, and *GLI1* transcript levels after infection of HMLE-shEcad cells with non-targeting (NT) or shRELA and shNFKB1 virus (shNFκB) virus. **(F)** Western blot of Gli1, p65, and p50 levels in HMLE-shEcad cells infected with either non-targeting (NT) or shRELA and shNFKB1 virus (shNFκB). GAPDH serves as a loading control. For all: * = *P* ≤0.05, *t* test. ** = *P* ≤0.005, *t* test. ChIP, chromatin immunoprecipitation; GLI1, glioma-associated oncogene 1; RELA, v-rel reticuloendotheliosis viral oncogene homolog A; NFKB1, nuclear factor of kappa light polypeptide gene enhancer in B-cells 1; NFκB, nuclear factor kappa-light-chain-enhancer of activated B cells; sh, short hairpin.

Since we observed these effects without stimulation or activation of the NFκB pathway, we hypothesized that NFκB is basally activated in our cells. Normally, NFκB subunits are sequestered in the cytoplasm due to inhibitory interactions with IκB. However, we observed that both the p65 and p50 subunits of NFκB present in the nucleus of EMT cells, while they are restricted to the cytoplasm of the HMLE control cell lines (Figure 7A). p65 and p50 show a similar localization pattern for claudin-low versus MCF10a cells (Figure S6 in Additional file 1). These data suggest that NFκB is basally present in the nucleus of EMT and claudin-low cells, and active even without stimulation by a proinflammatory agent, in agreement with some earlier reports describing constitutive NFκB activity in basal cell lines [50]-[52].

### NFκB binds to the GLIpromoter in EMT and claudin-low cells

The effects of triptolide on the expression levels of *GLI1* suggest that NFκB could act as a transcription factor to regulate *GLI1* expression. We searched the genomic region surrounding the transcription start site of *GLI1* for potential κB binding sites using SABiosciences DECipherment of DNA Elements (DECODE) system which searches for predicted binding sites of transcription factors using data from the ENCODE project consortium and the UCSC Genome Browser. Six potential κB binding sites were identified that matched the consensus κB binding sequence (Figure 7B). In order to determine if NFκB binds to the *GLI1* promoter we conducted ChIP experiments using primer sets designed to amplify each predicted binding site. Binding of the p65 subunit of NFκB to the first predicted κB binding site (chr12: 57851125–57851134) was enriched over the other binding sites in unstimulated HMLE-shEcad cells (Figure 7C). The binding of histone H3 to the six potential κB binding sites as well as to GAPDH serves as a positive control for the ChIP experiment (Figure 7C).

We also observed enrichment of p65 subunit binding to Site 1 in HMLE-Snail, BT549, and MDA.MB.436 cells (Figure S6B in Additional file 1). We did not detect enhanced binding of p65 subunits to Site 1 in the *GLI1* promoter of HMLE-shGFP or HMLE-pBP cell lines, despite observing enriched binding of histone H3 with Site 1, which serves as a positive control for the ChIP experiment (Figure S7A in Additional file 1). Overall the ChIP data suggest that there is crosstalk between NFκB and GLI1 signaling in EMT and claudin-low cell lines.

Since the NFκB inhibitor triptolide decreased the expression level of *GLI1*, we determined the impact of triptolide treatment on binding of p65 to the *GLI1* promoter. 1 μM triptolide treatment for six hours caused a 70% reduction in binding of p65 to Site 1 in the *GLI1* promoter of HMLE-shEcad cells compared to control vehicle treatment (Figure 7D). This supports the conclusion that triptolide decreases *GLI1* expression by inhibiting binding of NFκB transcriptional complexes to the *GLI1* promoter.

### Knockdown of NFκB results in decreased GLIexpression

Although triptolide has been shown to inhibit NFκB, it is not entirely specific. In order to reinforce the connection between NFκB and elevated *GLI1* levels, we determined the impact of NFκB knockdown on *GLI1* expression. Combined reduction of *RELA* of approximately 60%, and of *NFKB1* by 40% reduced *GLI1* expression at both the protein and transcript level in HMLE-shEcad cells (Figure 7E-F) and similar results were seen with knockdown of both NFκB subunits in MDA.MB.436 cells (Figure S7C in Additional file 1). Doxycycline-inducible knockdown of *RELA* of approximately 75% with two separate hairpins reduced *GLI1* expression (Figure S7D in Additional file 1). Hence, *GLI1* is regulated by NFκB in claudin-low and EMT cells.

## Discussion

Currently, there are no targeted treatment options for patients with claudin-low breast cancer, a particularly aggressive type of breast cancer. We used mammary carcinoma cell lines with induced EMT as surrogates for cells with stem-like characteristics and screened them for growth sensitivity to 150 targeted agents. Selective sensitivity of these cells to inhibition of GLI1 implicated *GLI1* as a vulnerable target. The transcriptional similarities of induced EMT mammary cells to claudin-low breast cancer suggested the potential importance of GLI1 for this breast cancer subset. Reduced *GLI1* expression impeded migration, clonogenicity, primary and secondary mammosphere formation and tumor formation by claudin-low breast cancer cells. These characteristics are associated with stem-like, invasive, and aggressive aspects of breast cancer, and suggest that inhibiting GLI1 may be an effective treatment strategy for patients with claudin-low breast cancer.

Our work reveals novel SMO-independent activation of *GLI1* by the NFκB pathway, in which the p65 subunit of NFκB binds directly to the *GLI1* promoter in EMT and claudin-low cells (Figure 7C). We have only observed binding of the p65 subunit to one κB binding site in the *GLI1* promoter, but this does not preclude binding of NFκB subunits to the remaining putative κB binding sites in the *GLI1* promoter, perhaps following cytokine stimulation. Knockdown of NFκB subunits resulted in decreased *GLI1* expression (Figure 7D-E), indicating transcriptional regulation of *GLI1* by NFκB. *GLI1* levels were not completely abrogated following knockdown of NFκB subunits, indicating either that residual NFκB activity is sufficient to sustain *GLI1* expression, or that other pathways contribute to *GLI1* transcription.

We also found that NFκB is activated through a non-canonical pathway in EMT and claudin-low cells. Typically, in the absence of an inflammatory signal NFκB dimers are sequestered in the cytoplasm by IκBα. However, we observed NFκB in the nucleus of EMT and claudin-low cells (Figure 7A and Figure S5 in Additional file 1) without stimulation, indicating that NFκB is present in an activated form in the nucleus. Interestingly, we observed less nuclear NFκB in claudin-low cell lines, which express less *GLI1*, notably HS578T and MDA.MB.231 cells. This association fits with our data indicating transcriptional regulation of *GLI1* by NFκB, and speaks to the molecular heterogeneity of the claudin-low subclass. Indeed, while claudin-low tumors express more *GLI1* than the basal, human epidermal growth factor receptor 2 (Her2), and luminal B subtypes overall, there still existed heterogeneity within this subset (Figure 2D). Activated NFκB [53] and expression of *GLI1*[17]-[19] have been associated with poor prognosis in breast cancer. It will be interesting to see if NFκB activity and *GLI1* expression are correlated in mammary tumors. Recently, nuclear *GLI1* expression was shown to be closely correlated with nuclear expression of NFκB in pancreatic cancer, and both were associated with shorter overall survival and worse outcome [54]. It will be interesting to determine if a similar phenomenon occurs in breast cancer, and if patients with tumors that co-express NFκB and *GLI1* have a worse outcome.

Constitutive activation of NFκB in nuclear lysates from breast cancer cells has been observed [55], and it will be interesting to determine the responsible factors that contribute to NFκB pathway activation in EMT and claudin-low cells. One possibility is ERBB3, since recent work has revealed that the ERBB3 ligand heregulin increases mammosphere formation in breast cancer cell lines, which was attenuated by NFκB pathway inhibition [56]. It will be interesting to explore the role of ERBB3 on NFκB in claudin-low cells and EMT, especially given our findings with EGFR in EMT cells (Figure S1 in Additional file 1) and the known interactions among ERBB family members in breast cancer [57]. Recently Yamamoto *et al*. identified activated NFκB in basal and claudin-low tumors, and a correlation between NFκB activity and JAG1 expression, which was associated with poor prognosis in the basal subset [52]. These results combined with our findings suggest that NFκB could affect different downstream targets depending on subtype, namely JAG1 in basal and GLI1 in claudin-low tumors.

Our work supports earlier studies implicating GLI1 signaling in some breast cancer cell lines, albeit through different mechanisms. *GLI1* expression is elevated in SUM1315 cells, and knockdown of *GLI1* in MDA.MB.231 cells reduces cell growth, invasion, and metastasis [41]. Targeting *GLI1* in inflammatory breast cancer has been shown to decrease the migratory ability of these cells, and to increase apoptosis [38]. We have extended these findings to the claudin-low subtype as a class, and our findings lend evidence to the potential of GLI1 as a therapeutic target in breast cancer.

There is increasing evidence for non-canonical Hh pathway activation in a variety of cancers [46] including breast [38],[42]. Recently, Goel *et al.* demonstrated a contribution of Neuropilin-2 (*NRP2*), a vascular endothelial growth factor (VEGF) co-receptor, to *GLI1* levels in claudin-low cell lines [40]. A non-canonical mechanism was implicated, since *SMO* knockdown did not interfere with the process. It will be of interest to determine if NRP2 exerts its effects on GLI1 through NFκB. Although the NFκB and Hh pathways are implicated in breast cancer, and these pathways share some common downstream targets [58], to our knowledge direct transcriptional crosstalk between the two pathways in breast cancer has not yet been reported.

Although our studies focused on *GLI1*, *GLI2* expression is also elevated in EMT and claudin-low cells (Figure 2A-B). While *GLI1* is itself a GLI1 regulatory target [34], in basal cell skin carcinoma cells, activation of *GLI2* by *GLI1* is indirect, and perhaps context dependent [59]. There are no consensus Gli or κB binding sites in the *GLI2* promoter. Therefore, it is likely that the regulation of *GLI2* expression in these cells occurs via a different mechanism than that described here for *GLI1*. Similarly, while some GLI1 targets have been identified [37], the activity of the Gli proteins is highly context dependent [34], and it will be of great interest to determine the effectors through which Gli1 mediates the biological phenotypes we observed in claudin-low lines.

EMT cells and claudin-low cells are closely related to cancer stem cells [5]-[7]. There is evidence for Hh signaling in normal and malignant human mammary stem cells, and upregulation of *GLI1* in mammospheres [16]. We observed upregulation of *GLI1* in mammospheres, and a decrease in primary and secondary sphere formation after *GLI1* knockdown (Figure 5C), strongly suggesting a role for *GLI1* in maintenance of breast cancer stem cells/progenitor cells. Elevated levels of *GLI1* transcripts were also seen in a published dataset of mammospheres grown from primary patient material (Figure 2D). We have shown here evidence of crosstalk between the GLI1 signaling and NFκB pathways in both EMT and claudin-low cell lines, indicating that activated *GLI1*, could be a mechanism that also operates in breast cancer stem cells. Recent reports substantiating the existence of cancer stem cells in solid tumors [60] reinforce the potential importance of these findings for breast cancer therapy.

## Conclusions

Several SMO antagonists are in clinical trials for the treatment of various cancers [61]. However, our findings indicate that, while bulk tumor and/or stromal cells may respond to SMO inhibitors, the tumor-initiating stem-like cells may not respond, possibly allowing for tumor resistance and recurrence. Crosstalk between these two key inflammatory and developmental pathways has important biological implications, and provides a rationale for combination therapy in the treatment of patients with claudin-low breast cancer. An increased molecular understanding of the signaling drivers in EMT and claudin-low cells may not only help patients with claudin-low or mesenchymal-like cancers, but could also aid in the prevention of metastasis and recurrence in breast cancer patients in general.

## Additional file

## Electronic supplementary material

Additional file 1: Table S1.: 150 compound screen. **Figure S1.** EMT cells have reduced EGFR and are resistant to EGFR inhibitors. **Figure S2.** *GLI2* and *GLI3* levels are not affected by JK184 treatment or by virus-expressing shGli1. **Figure S3.** Results of *GLI1* knockdown in control and basal cell lines. **Figure S4.** Biological replicate of *in vivo* experiment. **Figure S5.** Screen for upstream *GLI1* effectors. **Figure S6.** NFκB immunofluorescence in claudin-low cell lines. **Figure S7.** p65 ChIP from additional cell lines and additional knockdown experiments. (PDF 17 MB)

Below are the links to the authors’ original submitted files for images.

Authors’ original file for figure 1

Authors’ original file for figure 2

Authors’ original file for figure 3

Authors’ original file for figure 4

Authors’ original file for figure 5

Authors’ original file for figure 6

Authors’ original file for figure 7

## Abbreviations

ANOVA: analysis of variance
BSA: bovine serum albumin
CDH1: E-cadherin
ChIP: chromatin immunoprecipitation
DCIS: ductal carcinomas *in situ*
EGFR: epidermal growth factor receptor
EMT: epithelial-to-mesenchymal transition
FBS: fetal bovine serum
GAPDH: glyceraldehyde phosphate dehydrogenase
GLI1: glioma-associated oncogene 1
HER2: human epidermal growth factor receptor 2
Hh: Hedgehog
HMLE: human mammary epithelial cells, transformed with large T and small t antigens
NFKB1: nuclear factor of kappa light polypeptide gene enhancer in B-cells 1
NFκB: nuclear factor kappa-light-chain-enhancer of activated B cells
NRP2: neuropilin-2
NT: non-targeting
PBS(T): phosphate-buffered saline (with 0.1% Tween-20)
PCR: polymerase chain reaction
PTCH1: patched-1
RELA: v-rel reticuloendotheliosis viral oncogene homolog A
RT: room temperature
sh: short hairpin
SMO: smoothened
VEGF: vascular endothelial growth factor
