# Supplementary material for: Significance of glioma-associated oncogene homolog 1 (GLI1)expression in claudin-low breast cancer and crosstalk with the nuclear factor kappa-light-chain-enhancer of activated B cells (NFκB) pathway
Source: Breast Cancer Res. 2014 Sep 25;16:444. doi: 10.1186/s13058-014-0444-4 (PMC4303124; doi:10.1186/s13058-014-0444-4)

Figure Legends

Table S1. 150 agent screen. IC_{50} μM values for HMLE-shEcad and HMLE-shGFP cell lines are listed. Values were calculated from curves derived using XL-Fit software using Michaelis-Menten kinetics. NF indicates not determined, either because no curve fit could be obtained, or because the calculated IC_{50} was outside the experimental concentrations tested. Generally indicates lack of dose-dependent response to the agent tested. Agents representing standard of care therapy for triple negative breast cancer have been highlighted in bright yellow. Drugs which fall into similar classes of agents as those used in standard of care have been highlighted in pale yellow.

Figure S1. EMT cells have reduced EGFR and are resistant to EGFR inhibitors. **(A)** 384-well format dose-response curves of HMLE-shEcad (blue) and HMLE-shGFP (green) cells to erlotinib **(A)** or gefitinib **(B)**. Curve fit determined via XL-Fit using Michaelis-Menten kinetics. Red lines indicate IC_{50} value for HMLE-shGFP cells. Error bars indicate coefficient of variance between triplicate treatments. **(C)** Dose response curves generated in 96-well plate format with CellTiter-Glo for HMLE-shEcad (blue), HMLE-shGFP (green), HMLE-Snail (brown), and HMLE-pBP (gray) cells treated with gefitinib. Curve fit determined via XL-Fit using Michaelis-Menten kinetics. **(D)** Immunoblot of phosphorylated EGFR (pEGFR) and total EGFR in HMLE-shEcad and HMLE-shGFP cells. **(E)** Immunoblot of pEGFR and total EGFR in HMLE-shEcad and HMLE-shGFP cells treated with 10 ng/mL EGF for the indicated periods of time. Cells were starved in media lacking FBS for 5 hours prior to treatment. **(F)** Immunoblot of ERBB2 in HMLE-shEcad and HMLE-shGFP cells.

Figure S2. *GLI2* and *GLI3* levels are not affected by JK184 treatment or by virus expressing shGli1. Plot of *GLI2* **(A)** and *GLI3* **(B)** RNA levels in HMLE cells incubated

with JK184 at the indicated concentrations for three days. Plot of *GLI2* **(C)** and *GLI3* **(D)** transcript levels in claudin-low cell lines treated with the indicated JK184 doses for three days. Plot of *GLI2* **(E)** and *GLI3* **(F)** transcript levels in claudin-low cell lines infected with retroviruses targeting the indicated transcripts.

Figure S3. Results of *GLI1* knockdown in control and basal cell lines. **(A)** Relative transcript levels of claudin-low (BT549, HBL100, HS578T, MDA.MB.157, MDA.MB.231 and MDA.MB.436), basal (HCC1806), and control (MCF10a, MTSV1-7) cell lines. **(B)** Western blot and **(C)** real-time data depicting efficacy of *GLI1* knockdown in HCC1806 and MTSV1-7 cell lines. **(D)** Colony formation, **(E)** migration, **(F)** proliferation, and **(G)** sphere forming ability of the indicated cell lines with either a non-targeting (NT) or sh*GLI1*-targeted (sh*GLI1* #1) retrovirus. Cells were selected with puromycin for three days prior to experimentation. **(H)** Plot of *GLI1* expression levels in adherent HCC1806 cells compared to cells grown as spheres.

Figure S4. Biological replicate of *in vivo* experiment. **(A)** Plot of tumor volume over time arising from orthotopic injection of MDA.MB.436 cells infected with either non-targeting (NT) or *GLI1* knockdown constructs. n = four animals.

Figure S5. Screen for upstream *GLI1* effectors. **(A)** Candidate agents were grouped into four pools, with 1 μM of each drug used for treatment. DMSO is the vehicle control, and JK184 (1 μM) a positive control for Gli1 suppression. Real-time RT-PCR analysis for *GLI1* mRNA was conducted after 16h treatment. **(B)** Agents from groups 3 and 4 were analyzed individually for their effects on *GLI1* transcript levels, as in A). **(C)** NFκB reporter assay showing effects of triptolide on κB reporter assay (firefly) compared to

control (renilla) luciferase activity. The luciferase reporter (pBII-Luc) and pRL-TK renilla control plasmids were transfected into cells using X-tremeGENE 9 (Roche). 16h after transfection the medium was replaced with growth medium. The following day, cells were treated with 1 μM triptolide or DMSO control for 6 hours. Cells were assayed using the Dual-Luciferase Reporter Assay Reporter System (Promega).

Figure S6. NFκB immunofluorescence in claudin-low cell lines. Immunofluroscence images taken at 200x magnification showing localization of NFκB p50 (left-most panels, I) or p65 (left-most panels, II) subunits in claudin-low or MCF10a cell lines. DAPI staining indicates nuclei. Scale bar indicates 100 μm.

Figure S7. p65 ChIP from additional cell lines and additional knockdown experiments. **(A)** ChIP experiment conducted in HMLE-shGFP and HMLE-pBP cell lines. Binding to Site 1 in the *GLI1* promoter is depicted for pull-downs with control IgG or antibody directed against p65 or histone H3. **(B)** Graph depicting the relative fold enrichment of p65 binding to Site 1 compared to a control site (GAPDH). Error bars indicate standard error derived from two experiments. **(C)** Knockdown of *RELA* and *NFκB1* in MDA.MB.436 cells, and effect on transcript levels of *RELA*, *NFkB1*, and *GLI1*. Error bars are the standard error of the mean between three biological replicates.*= p-value below 0.05, ** = p-value below 0.005. **(D)** Inducible knockdown of *RELA* in MDA.MB.436 cells, and effect on transcript levels of *RELA*, *NFkB1*, and *GLI1*. Error bars are the standard error of the mean between three biological replicates.*= p-value below 0.05, ** = p-value below 0.005.

Table S1. 150 compound screen. IC50 (μM) values for HMLE-shEcad and HMLE-shGFP cell lines are listed.

Compound	shGFP	shEcad
10058-F4	2.82	NF
17-AAG	0.06	0.11
17-DMAG	0.01	0.04
2-Deoxy-D-glucose	NF	NF
4'Z D4T	NF	NF
8-1-T	NF	NF
ABT-737	NF	NF
ABT-888	NF	NF
AG490	NF	NF
AG538	NF	NF
Akt inhibitor III	NF	NF
AR-A014418	NF	NF
Arsenic trioxide	NF	NF
Axitinib	NF	NF
AZD 7762	0.09	0.03
B8	NF	NF
Bay11-7085	2.38	2.64
Bexarotene	NF	NF
BEZ-235	0.01	0.09
BIBR-1532	NF	NF
Bithionol	NF	NF
BMS-536924	NF	NF
Bortezomib	0.01	0.01
Bosutinib	0.37	>10
BQ 788	NF	NF
Bromopyruvic acid	NF	NF
Bryostatin 1	NF	NF
BX 513 hydrochloride	9.32	11.29
Capecitabine	NF	NF
Carboplatin	NF	NF
Carmustine	NF	NF
Celecoxib	NF	NF
Cerulenin	NF	NF
CID 755673	NF	NF
CIP 13-74	1.16	1.42
CIP-1359	0.75	4.13
Cisplatin	NF	NF

Curcumin	6.45	10.94
CW3	NF	NF
Cyclopamine	NF	NF
Cytarabine HCl	0.29	1.44
Dasatinib	0.08	0.55
Daunorubicin HCl	0.04	0.06
Decitabine	NF	NF
Dehydroepiandrosterone (DHEA)	NF	NF
Dibenzazepine (DBZ)	NF	NF
Disulfiram	NF	NF
Dovitinib	0.54	5.61
Doxorubicin	0.03	0.09
EGCG	NF	NF
Embelin	NF	NF
Enzastaurin	NF	NF
Eriocalyxin B	0.34	0.19
Erlotinib	0.24	>10
Etoposide	1.07	1.93
Everolimus	NF	NF
FAK Inhibitor 14	0.80	0.85
Flavopiridol	0.13	0.31
FTI 276	NF	NF
GDC 0449	NF	NF
GDC 0879	NF	NF
Gefitinib	0.09	NF
GW 9662	NF	NF
GW5074	NF	NF
HA14-1	NF	NF
Hydroxychloroquine sulfate	5.41	14.95
Imatinib	>10	NF
Imiquimod	NF	NF
Irinotecan HCl	3.61	7.04
Ixabepilone	0.00	0.00
JK184	0.01	0.00
JNK inhibitor II	NF	NF
Lapatinib	0.34	NF
LFMAU	NF	NF
LOddC	0.71	4.73
Ly294002	NF	NF
Melphalan	NF	NF
Methotrexate	0.02	0.01
MIF-098	NF	NF

MIF-103	NF	NF
MIF-108	NF	NF
MIF-112	NF	NF
MIF-139	NF	NF
MIF-153	NF	NF
MIF-154	NF	NF
Mitomycin C	0.46	1.44
MK-2206	4.42	NF
Nilotinib	NF	NF
NSC 625987	NF	NF
NSC 66811	NF	NF
Nutlin-3	NF	NF
NV128	0.28	0.23
NV356	0.75	0.57
NV360	0.72	0.45
Obatoclax	0.26	0.26
Onrigin	NF	NF
OPC-32	NF	NF
Oxaliplatin	NF	NF
Paclitaxel	0.00	0.01
PD-0332991	7.30	NF
PD173074	2.74	4.00
PD198306	NF	NF
Pentostatin	NF	NF
Perifosine	0.66	2.95
PHA 665752	NF	NF
PLX 4032	NF	NF
PLX 4720	NF	NF
PNU 74654	NF	NF
PP2	NF	NF
PQ401	NF	NF
PRIMA-1	NF	NF
PX 12	7.11	5.69
Rapamycin	NF	NF
Roscovitine	NF	NF
S31-201	NF	NF
S6	NF	NF
Salermide	NF	NF
SANT-2	NF	NF
SB-203580	NF	NF
SB-431542	NF	NF
Simvastatin	7.58	7.34

SL 0101-1	NF	NF
Sodium Dichloroacetate	NF	NF
Sodium stibogluconate	NF	NF
Sorafenib	6.49	9.50
Stattic	0.96	0.96
Staurosporine	0.00	0.00
SU5402	NF	NF
Sunitinib	2.64	7.39
Syk Inhibitor	NF	NF
Tamoxifen citrate	NF	NF
Temozolomide	NF	NF
Temsirolimus	NF	NF
Thalidomide	NF	NF
Topotecan	0.05	0.13
Tozasertib	1.21	4.31
Tretinoin	NF	NF
Triapine	0.68	0.93
Trichostatin A	0.21	0.35
Trilophorin	NF	NF
Triptolide	0.00	0.00
Troglitazone	NF	NF
Tylophorin	0.38	0.39
U0126	NF	NF
Vandetanib	5.01	NF
Vatalanib	NF	NF
Vinblastine sulfate	0.01	0.01
Vorinostat	1.68	1.50
XAV 939	NF	NF
Y-27637	NF	NF

Figure S1: EMT cells have reduced EGFR and are resistant to EGFR inhibitors

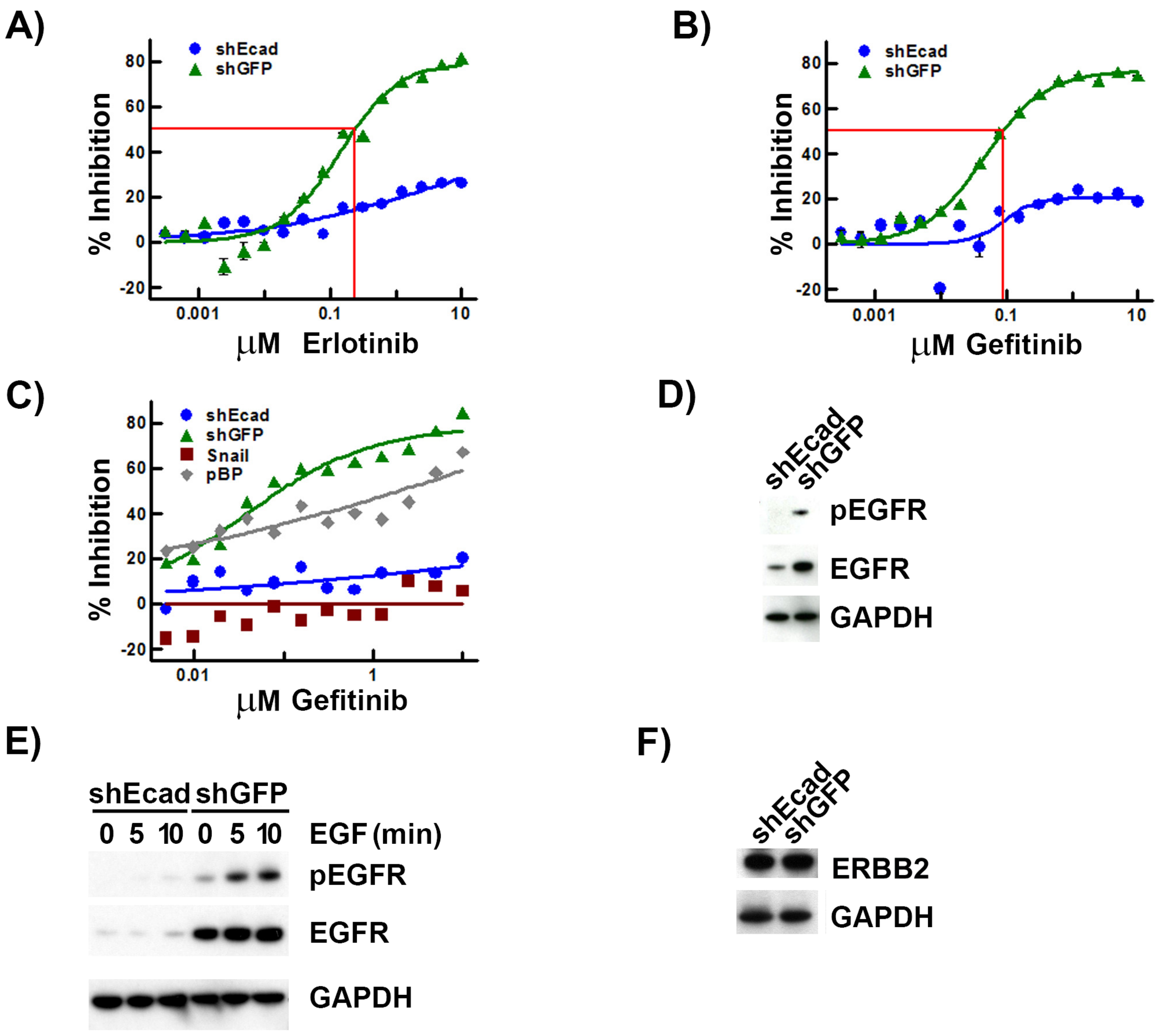

Figure S2: *GLI2* and *GLI3* levels are not affected by JK184 treatment or by virus expressing sh*GLI1.*

A)

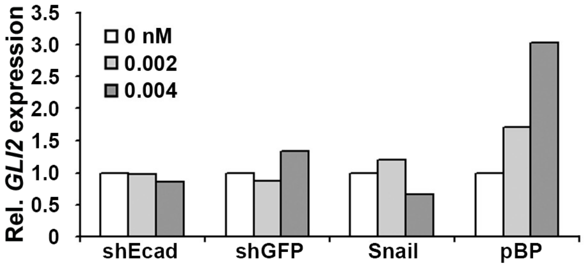

B)

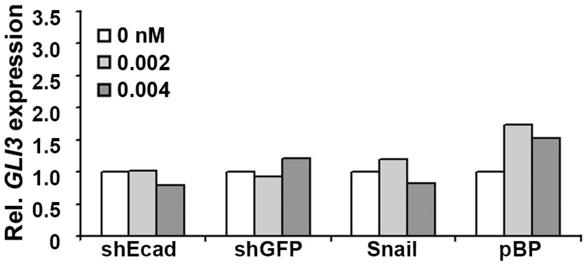

C)

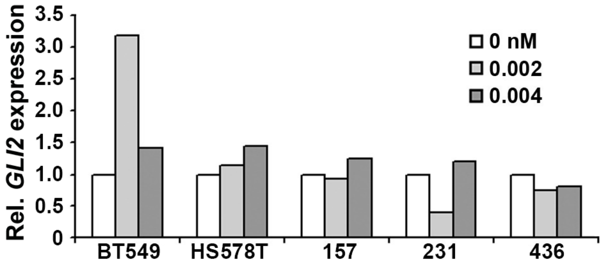

D)

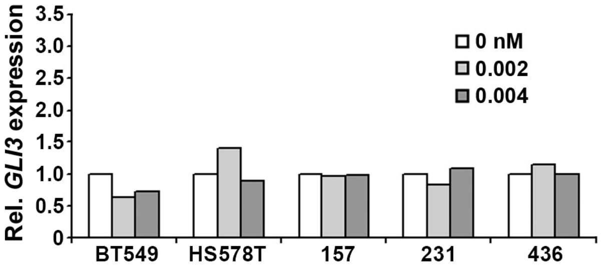

E)

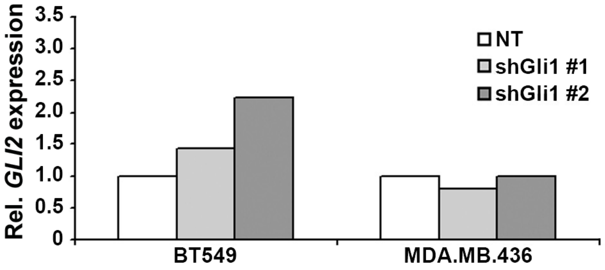

F)

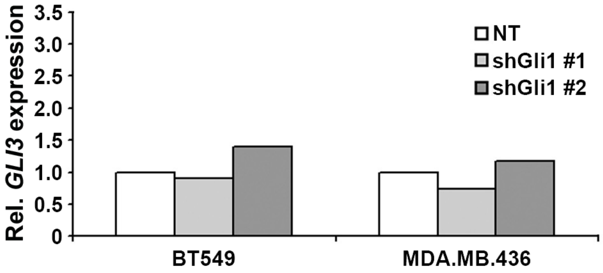

Figure S3: Results of *GLI1* knockdown in control and basal cell lines.

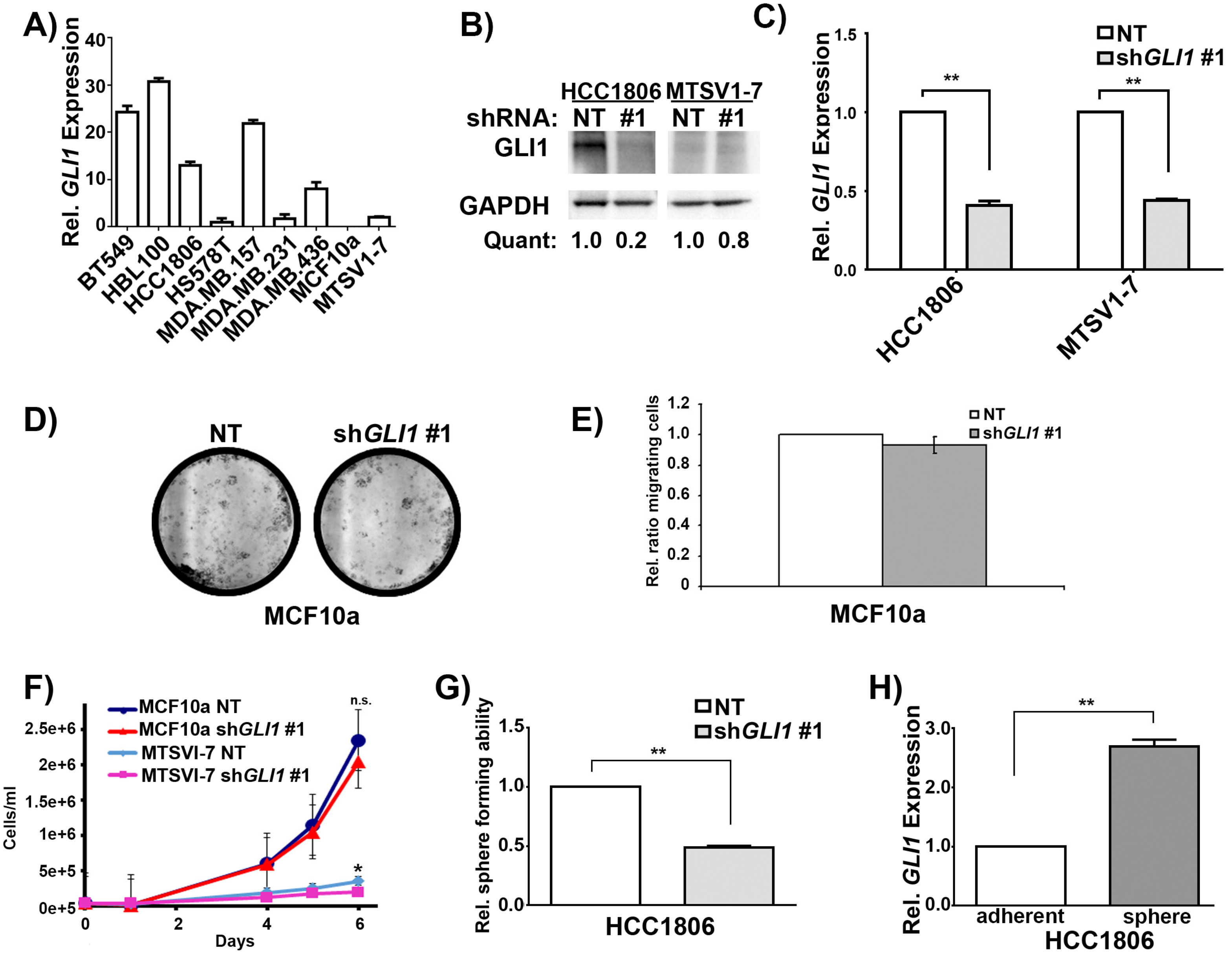

Figure S4: Biological replicate of *in vivo* experiment.

A)

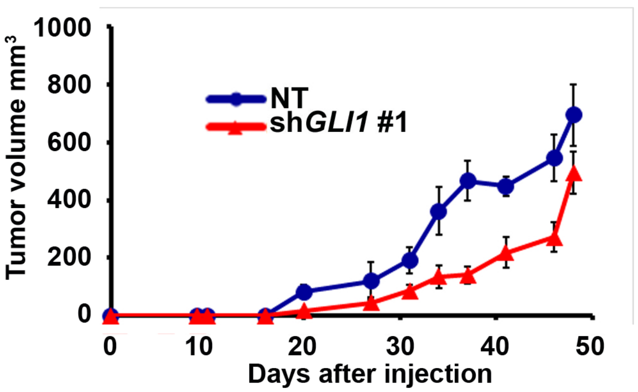

Figure S5: Screen for upstream *GLI1* effectors.

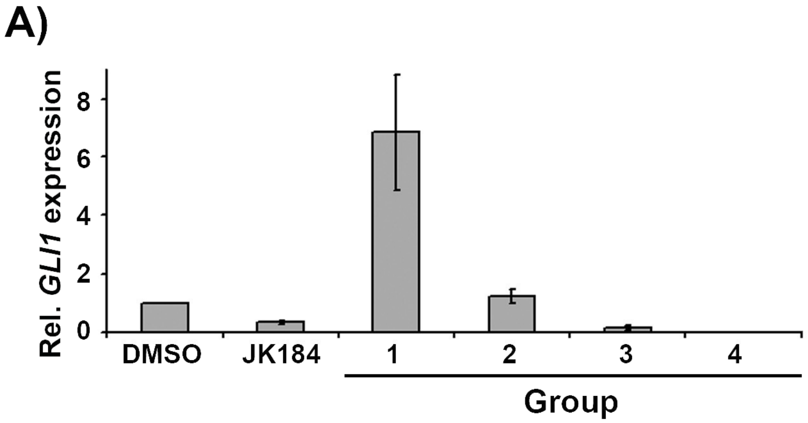

Group 1	Group 2	Group 3	Group 4
MK2206	Bez-235	Bosutinib	AR-A014413
Sunitinib	Obatoclax	SB203530	Erlotinib
U0126	Pha665752	Triptolide	JNK Inhib. II
			Stattic

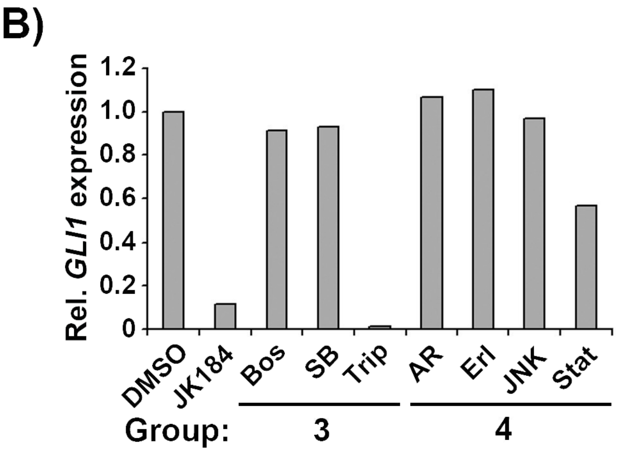

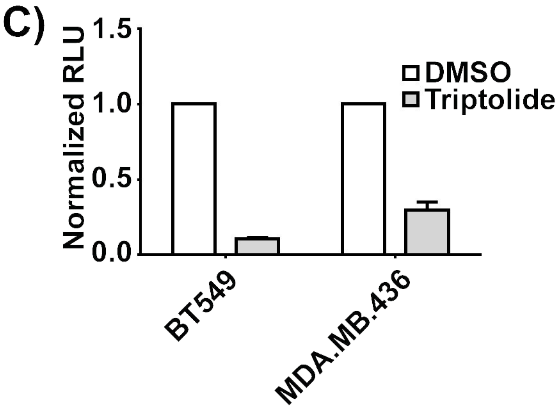

Figure S6: NFκB immunofluorescence in claudin-low cell lines.

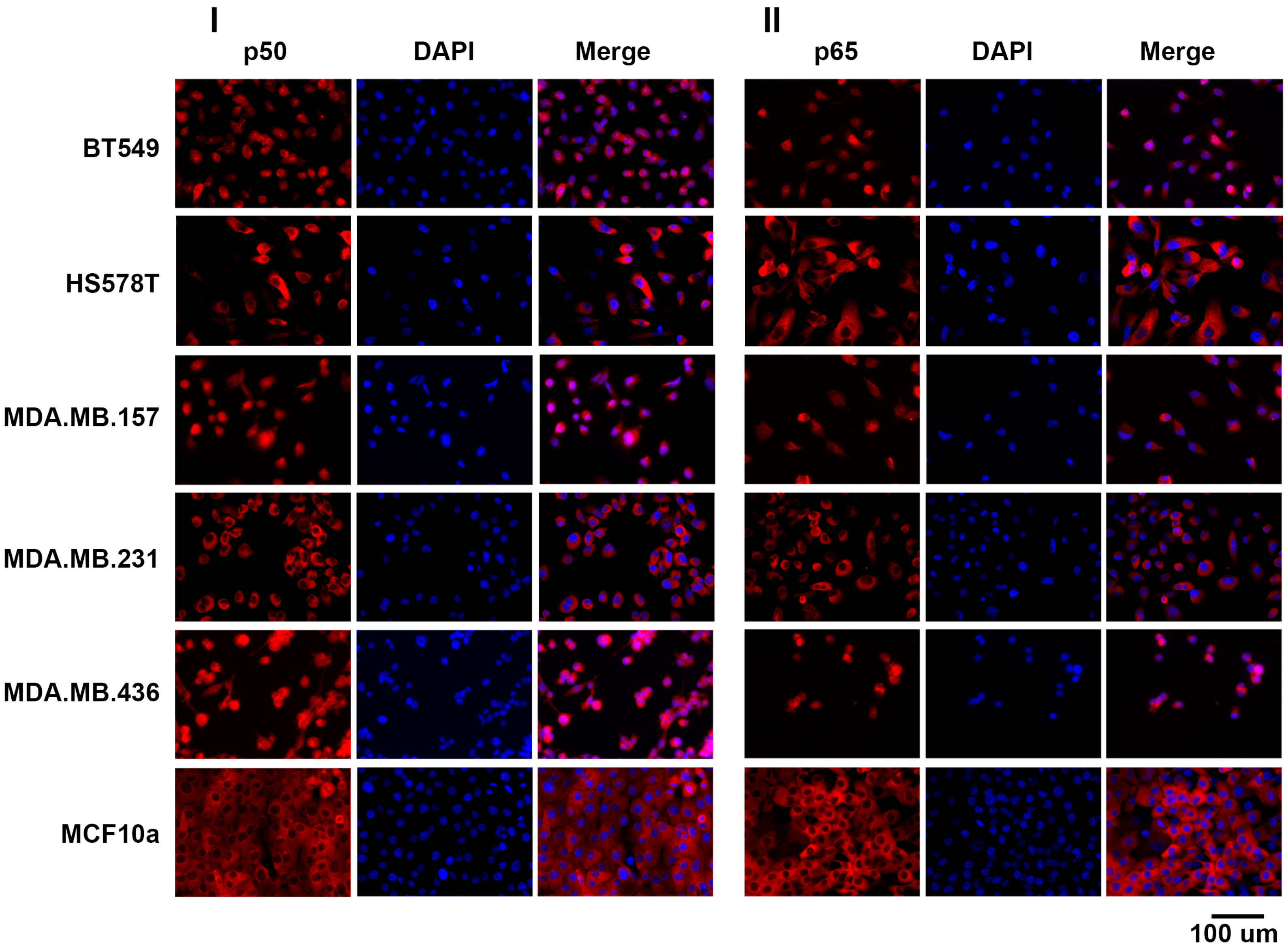

Figure S7: p65 ChIP from additional cell lines and additional knockdown experiments

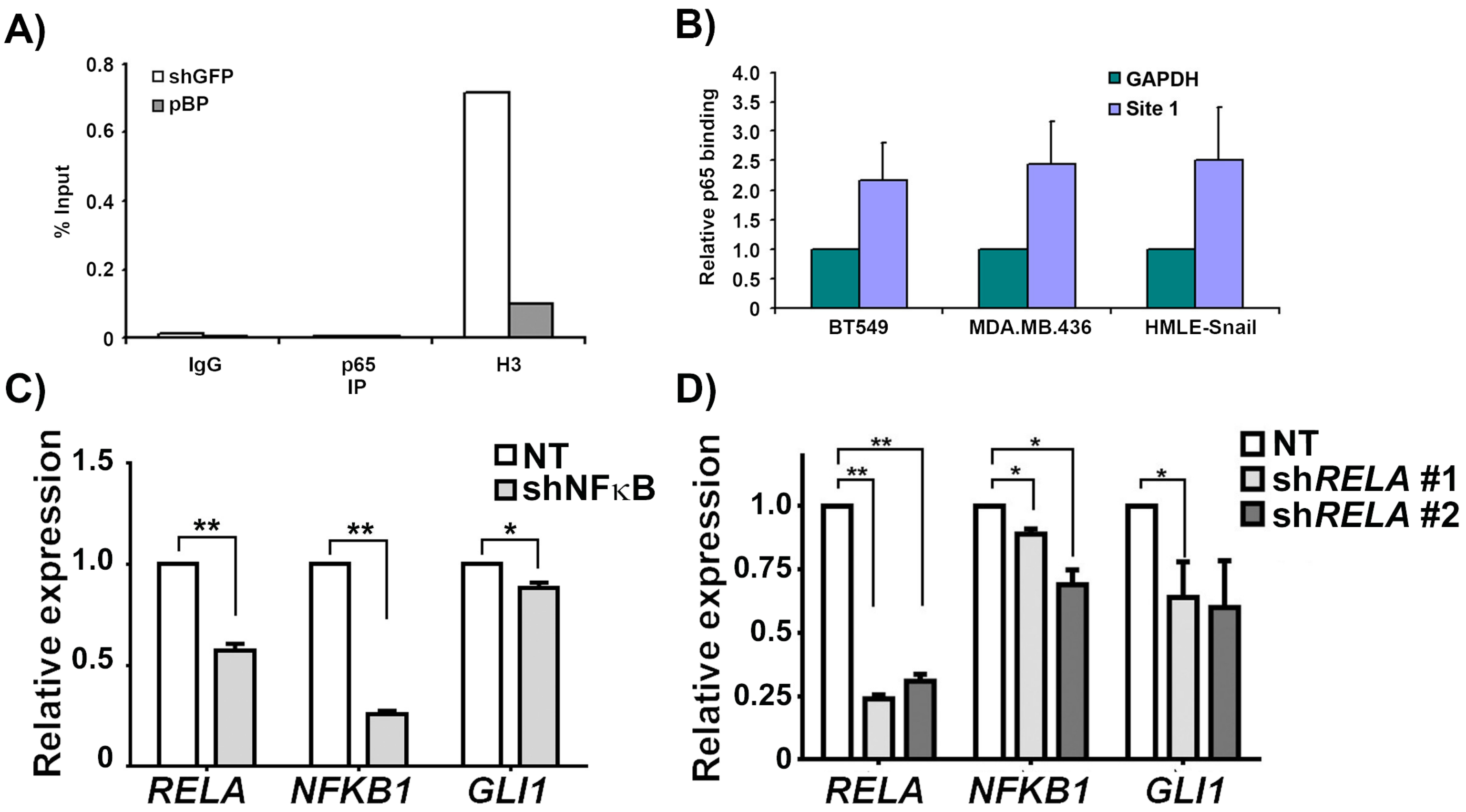

Supplement: Supplementary file 1 — Additional file 1: Table S1.: 150 compound screen. Figure S1. EMT cells have reduced EGFR and are resistant to EGFR inhibitors. Figure S2. GLI2 and GLI3 levels are not affected by JK184 treatment or by virus-expressing shGli1. Figure S3. Results of GLI1 knockdown in control and basal cell lines. Figure S4. Biological replicate of in vivo experiment. Figure S5. Screen for upstream GLI1 effectors. Figure S6. NFκB immunofluorescence in claudin-low cell lines. Figure S7. p65 ChIP from additional cell lines and additional knockdown experiments. (PDF 17 MB) [file 13058_2014_444_MOESM1_ESM.pdf]
