# Supplementary figures and images for: Significance of glioma-associated oncogene homolog 1 (GLI1)expression in claudin-low breast cancer and crosstalk with the nuclear factor kappa-light-chain-enhancer of activated B cells (NFκB) pathway
Source: Breast Cancer Res. 2014 Sep 25;16:444. doi: 10.1186/s13058-014-0444-4 (PMC4303124; doi:10.1186/s13058-014-0444-4)

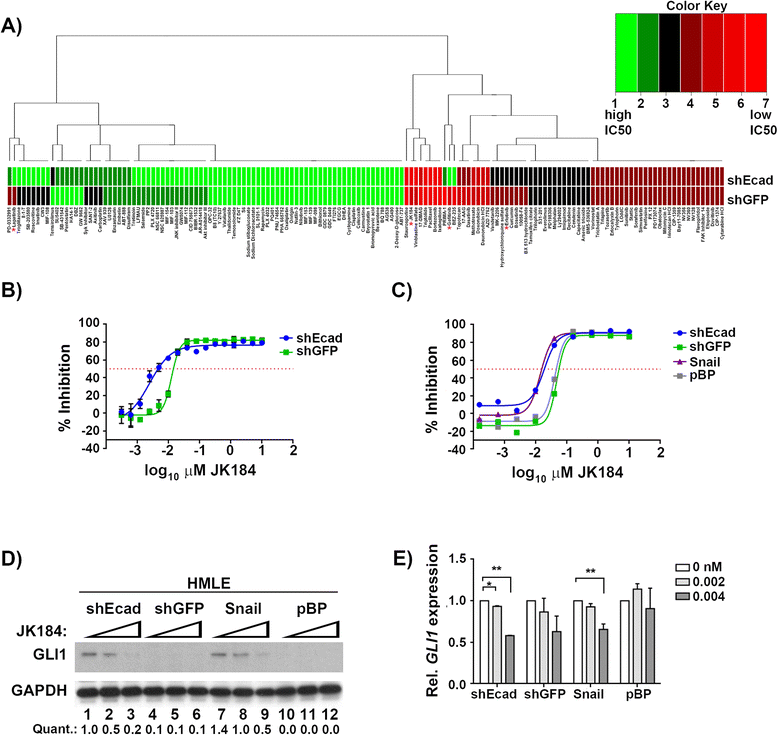

Supplement: Supplementary file 2 — Authors’ original file for figure 1 [file 13058_2014_444_MOESM2_ESM.gif]

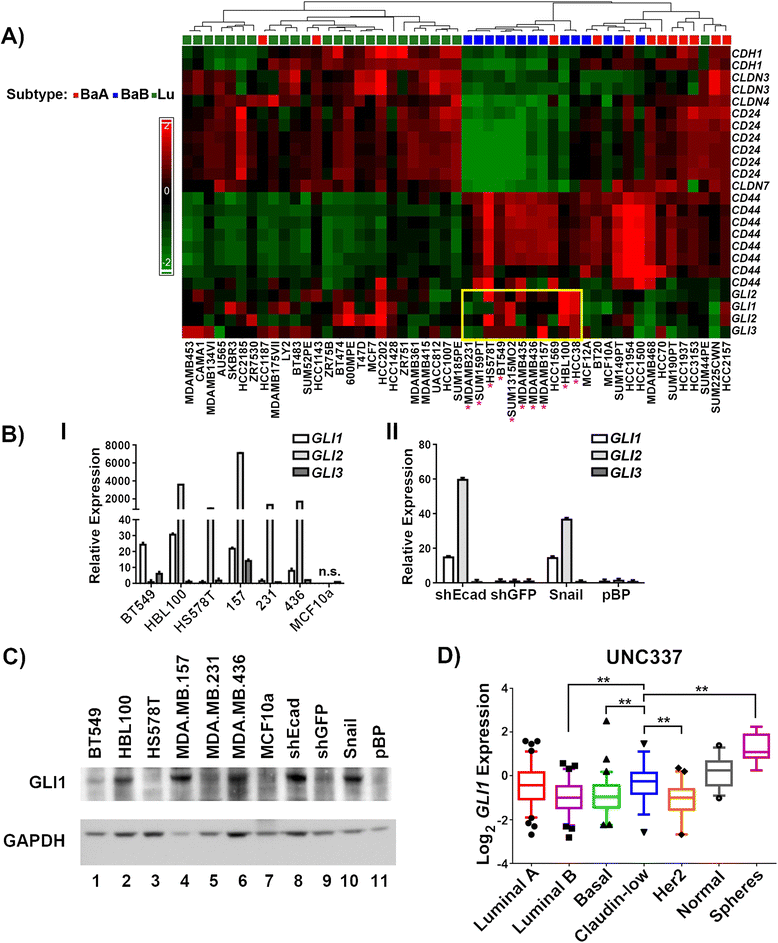

Supplement: Supplementary file 3 — Authors’ original file for figure 2 [file 13058_2014_444_MOESM3_ESM.gif]

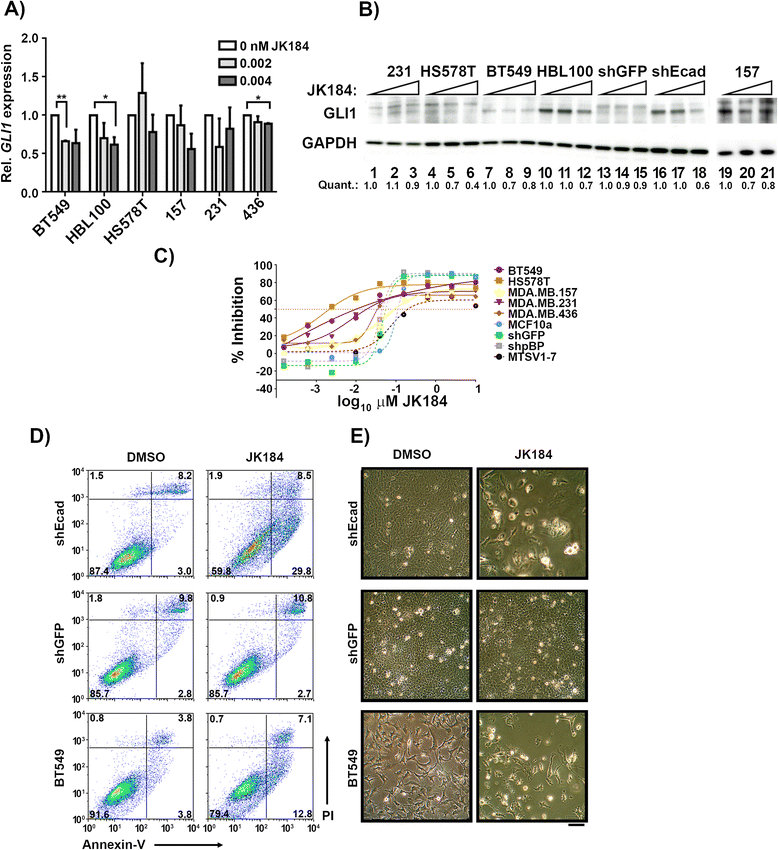

Supplement: Supplementary file 4 — Authors’ original file for figure 3 [file 13058_2014_444_MOESM4_ESM.gif]

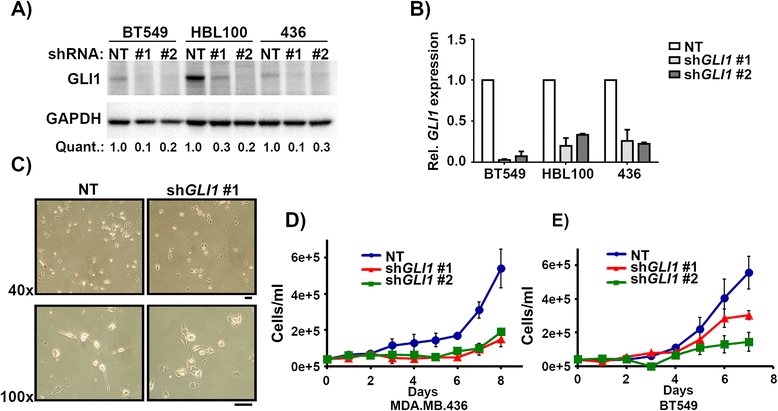

Supplement: Supplementary file 5 — Authors’ original file for figure 4 [file 13058_2014_444_MOESM5_ESM.gif]

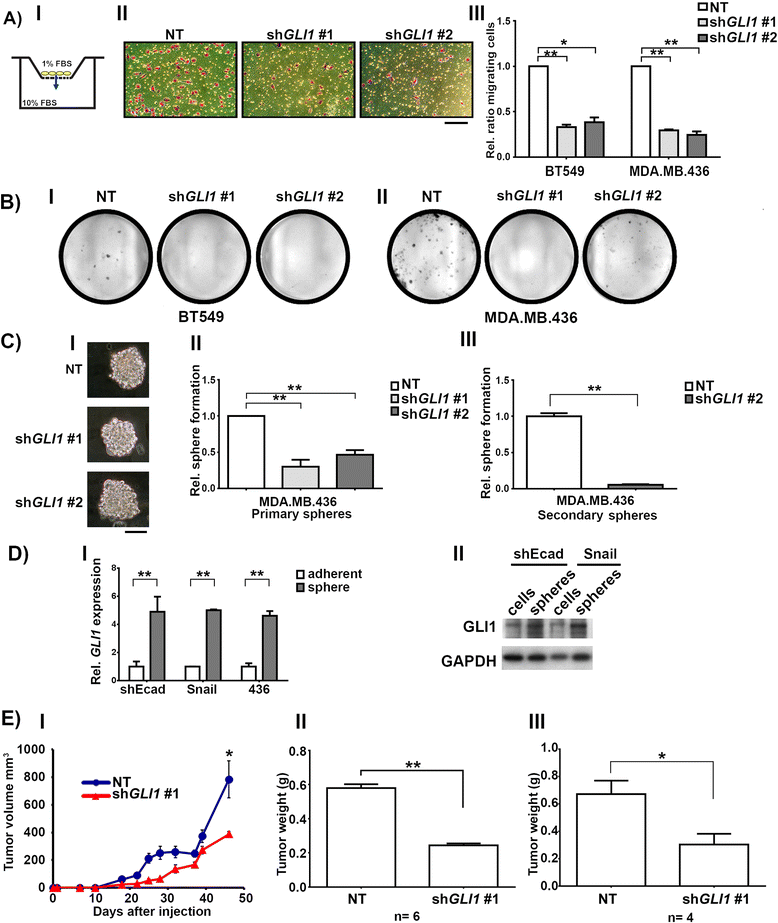

Supplement: Supplementary file 6 — Authors’ original file for figure 5 [file 13058_2014_444_MOESM6_ESM.gif]

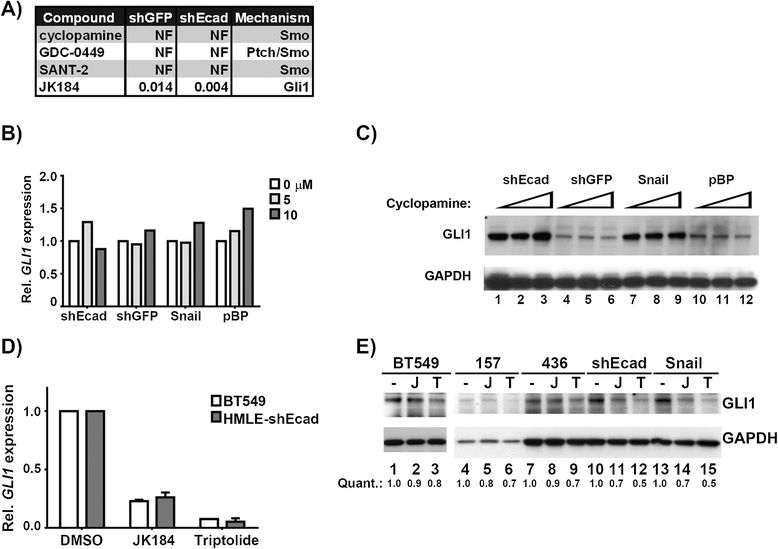

Supplement: Supplementary file 7 — Authors’ original file for figure 6 [file 13058_2014_444_MOESM7_ESM.gif]

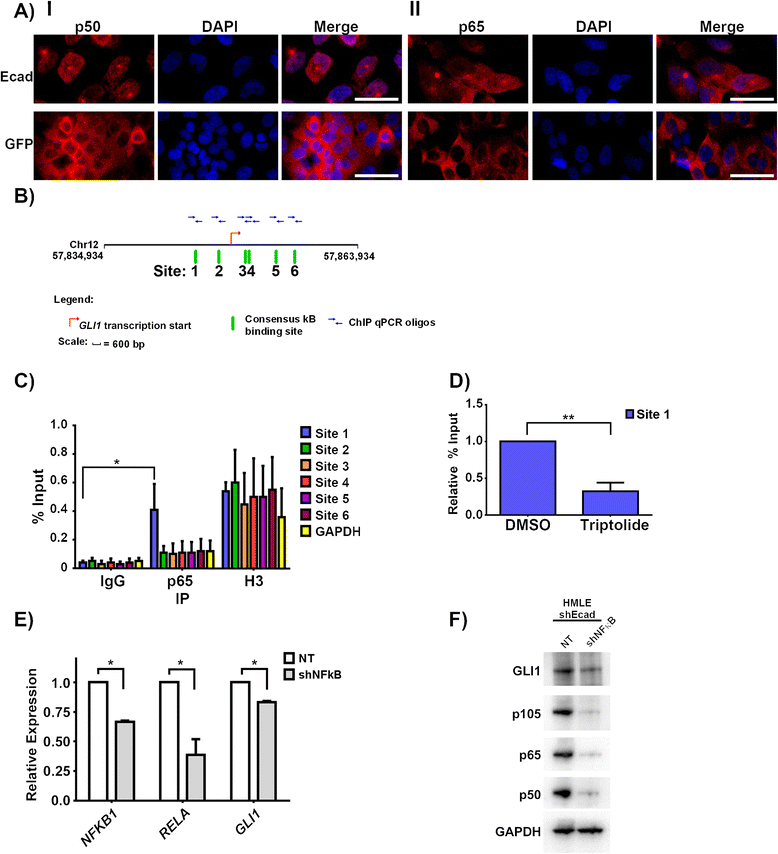

Supplement: Supplementary file 8 — Authors’ original file for figure 7 [file 13058_2014_444_MOESM8_ESM.gif]
